# Supplementary material for: Immobilized Sulfuric Acid on Silica Gel as Highly Efficient and Heterogeneous Catalyst for the One-Pot Synthesis of Novel α-Acyloxycarboxamides in Aqueous Media
Source: Int J Mol Sci. 2022 Aug 23;23(17):9529. doi: 10.3390/ijms23179529 (PMC9455172; doi:10.3390/ijms23179529)
Supplement: Supplementary file 1 [file ijms-23-09529-s001.zip › ijms-1872740-supplementary.pdf]

Immobilized Sulfuric Acid on Silica Gel as Highly Efficient and Heterogeneous Catalyst for the One-Pot Synthesis of Novel α-Acyloxycarboxamides in aqueous media.

Sodeeq Aderotimi Salami[1,*], Meloddy Manyeruke [1], Xavier Siwe-Noundou [2] and Rui Werner Maçedo Krause [1,*]

1 Department of Chemistry, Rhodes University, Grahamstown 6140, South Africa

2 Department of `Pharmaceutical Sciences, School of Pharmacy, Sefako Makgatho Health Sciences University, Pretoria 0204, South Africa

* Correspondence: sodeqaderotimi@gmail.com (S.A.S.); r.krause@ru.ac.za (R.W.M.K.);
Tel.: +27-83-302-3511 (S.A.S.); +27-46-603-7030 (R.W.M.K.)

Abstract: Application of immobilized sulfuric acid on silica gel as an efficient and easily reusable solid catalyst has been explored in the synthesis of novel α-acyloxycarboxamide derivatives via Passerini reaction of benzoic acid, aldehyde/ketone, and isocyanides. The passerini adducts were obtained in high to excellent yields within 10 minutes in aqueous media under catalytic condition. The key advantages of the process include short reaction time, high yields, the catalyst's low cost, and the catalyst's reusability (H_2SO_4-SiO_2).

Keywords: Passerini reaction; aqueous media; α-acyloxycarboxamides; immobilized sulfuric acid on silica gel.

NMR spectral data of synthesised compounds.

A. (2-nitrophenylcarbamoyl)(phenyl)methyl benzoate.

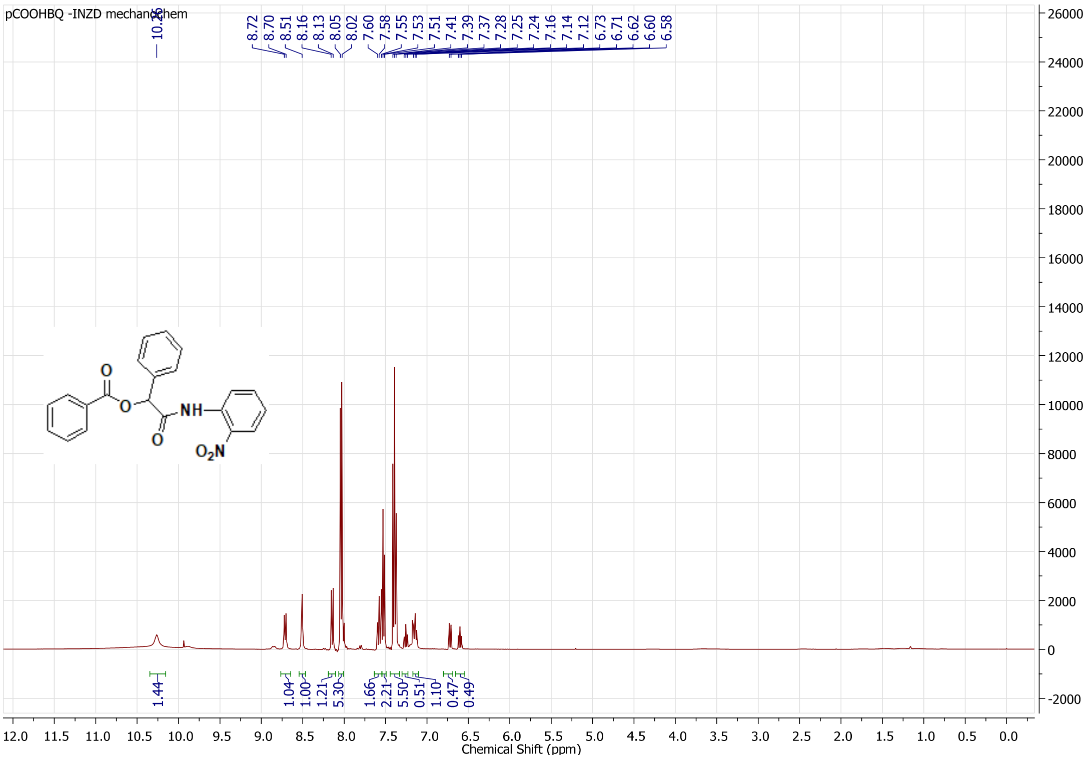

Figure S1.1. ^{1}H NMR (400 MHz, $CDCl_3$) spectrum

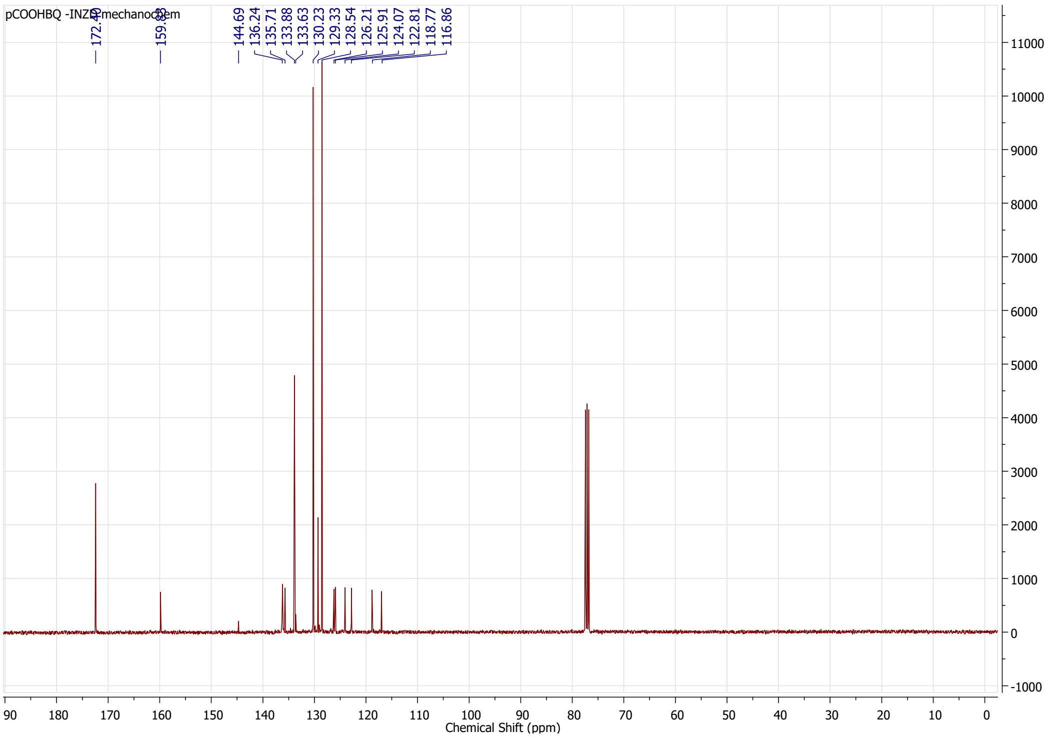

Figure S1.2. ^{13}C NMR (101 MHz, $CDCl_3$) spectrum

B. (2-bromophenylcarbamoyl)phenyl)methyl benzoate,

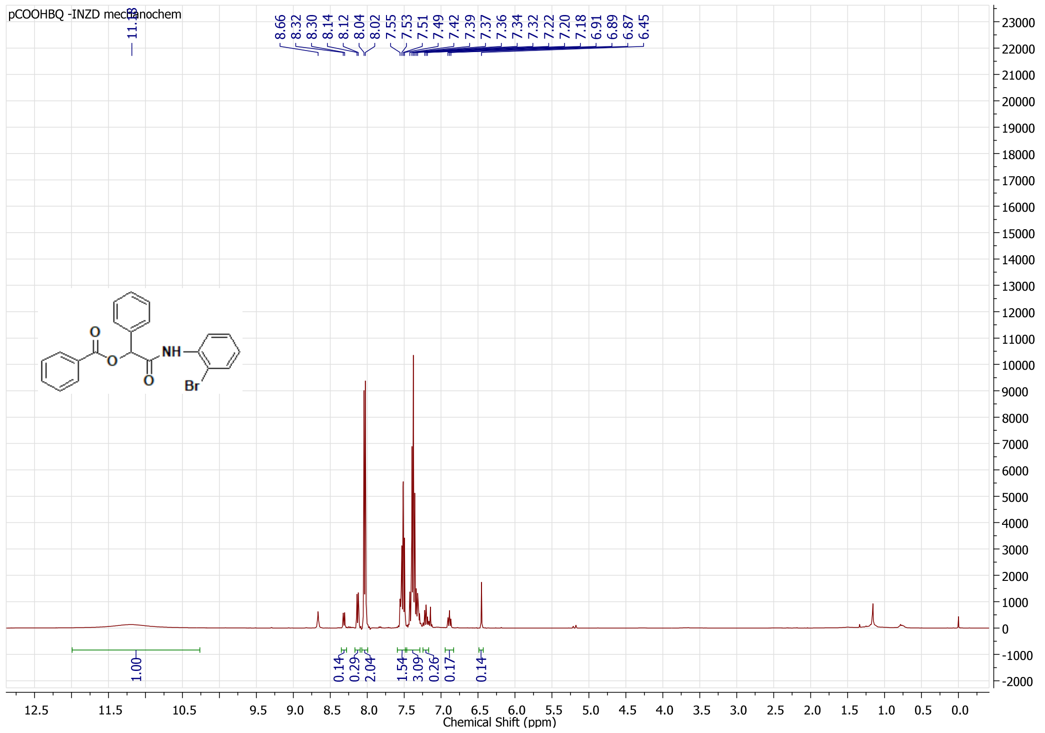

Figure S2.1. ^{1}H NMR (400 MHz, $CDCl_3$) spectrum

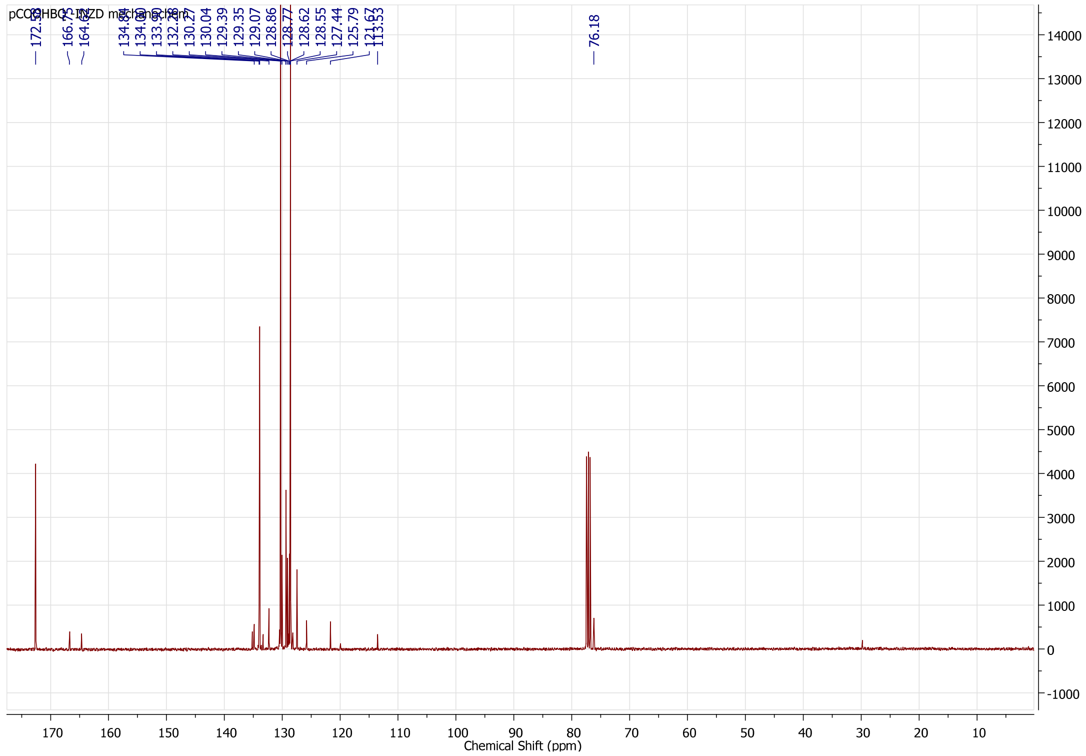

Figure S2.2. ^{13}C NMR (101 MHz, $CDCl_3$) spectrum

C. (2-chlorophenylcarbamoyl)(phenyl)methyl benzoate,

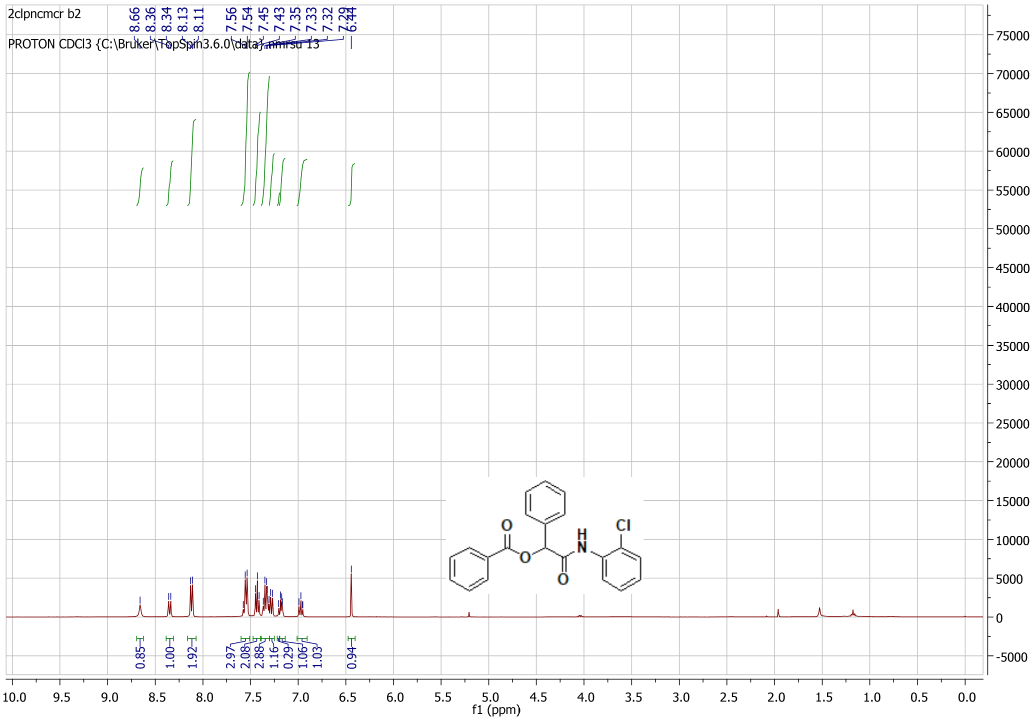

Figure S3.1. ^{1}H NMR (400 MHz, $CDCl_3$) spectrum

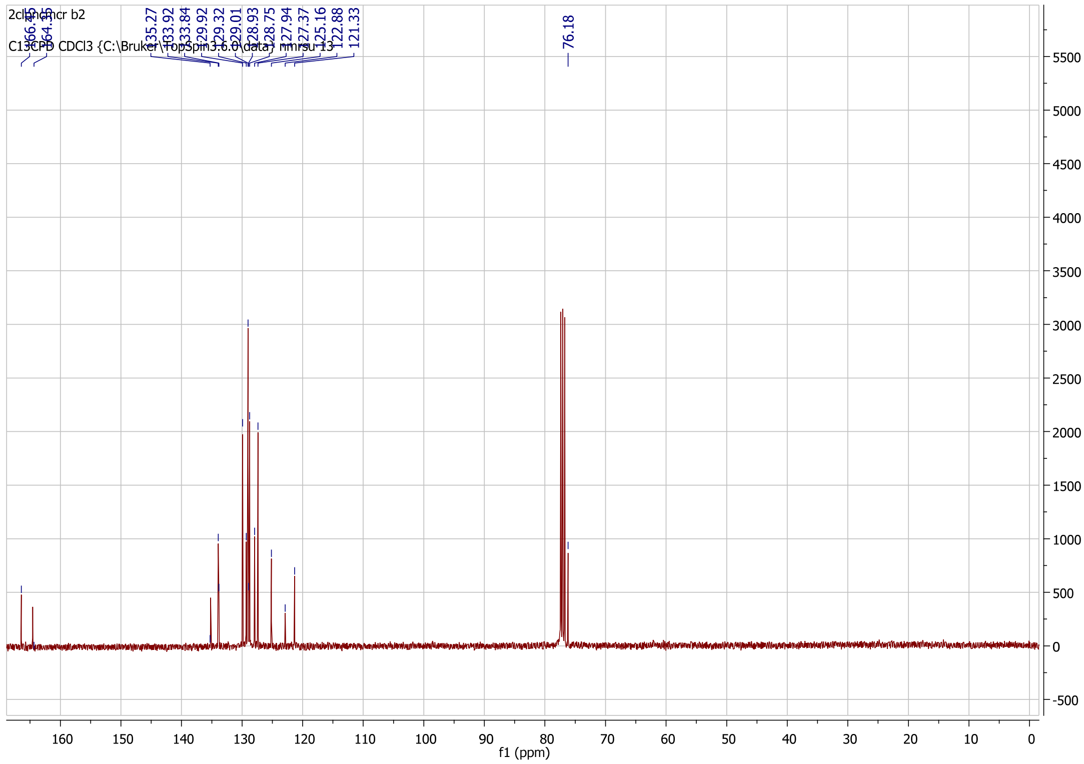

Figure S3.2. ^{13}C NMR (101 MHz, $CDCl_3$) spectrum

C. (3,4-difluorophenylcarbamoyl)(phenyl)methyl benzoate,

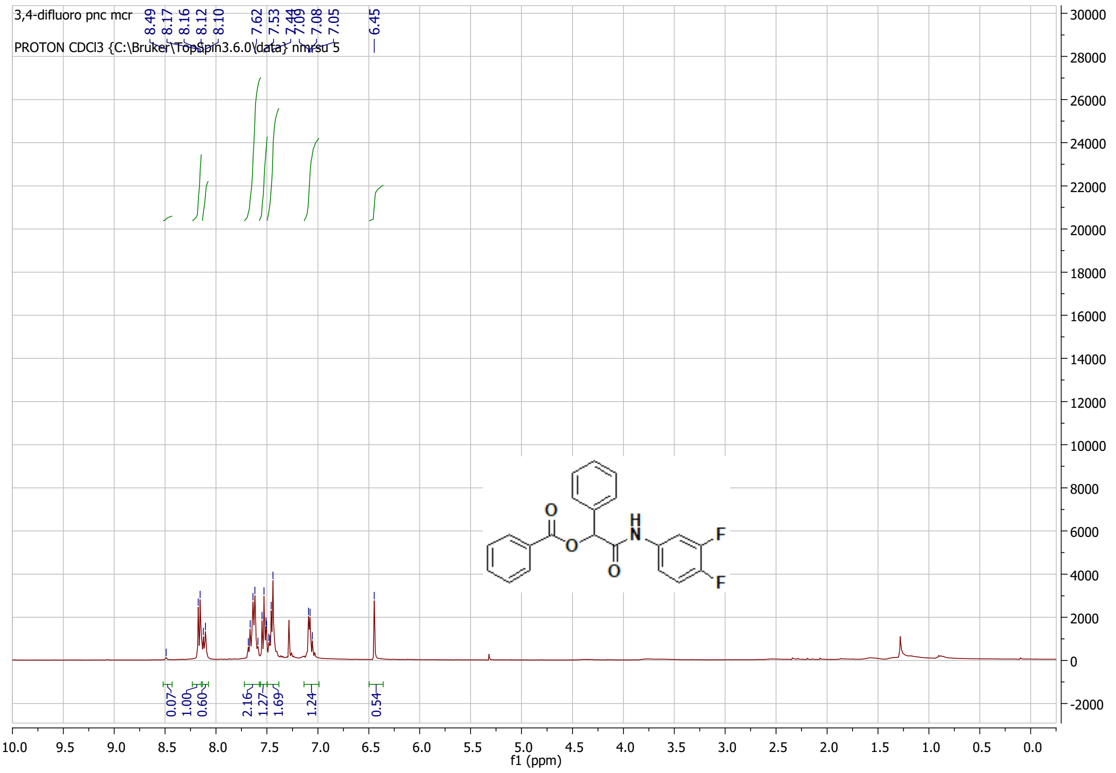

Figure S4.1. ^{1}H NMR (400 MHz, $CDCl_3$) spectrum

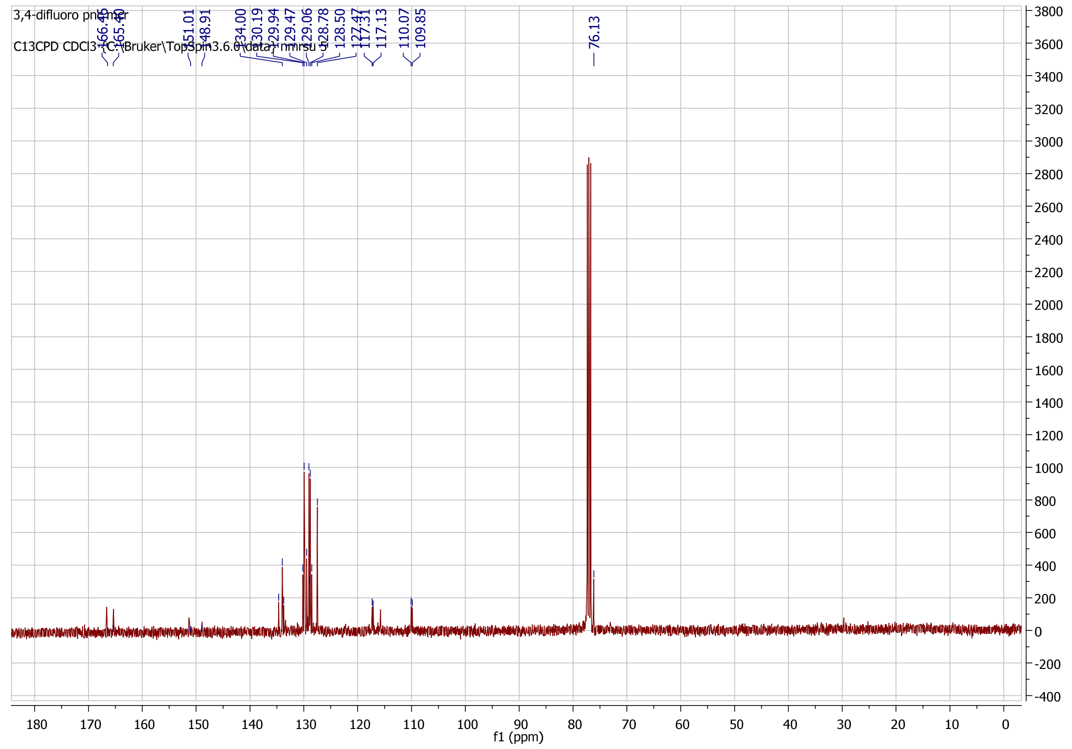

Figure S4.2. ^{13}C NMR (101 MHz, $CDCl_3$) spectrum

D. (3,4-dichlorophenylcarbamoyl)phenyl)methyl benzoate,

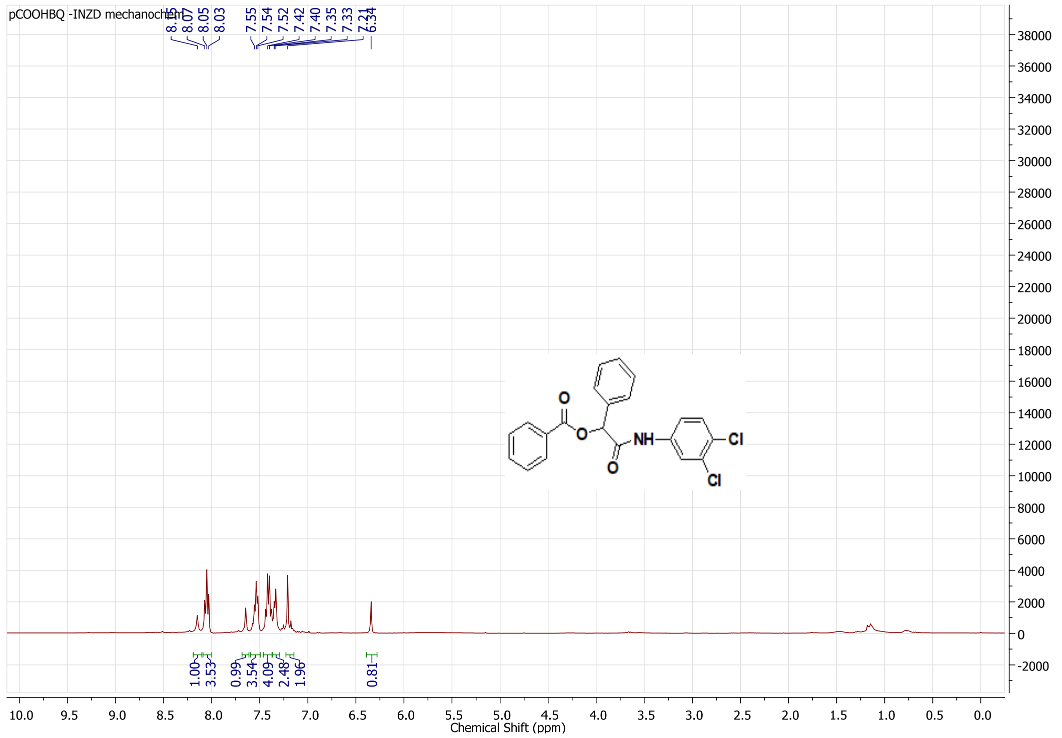

Figure S5.1. ^{1}H NMR (400 MHz, $CDCl_3$) spectrum

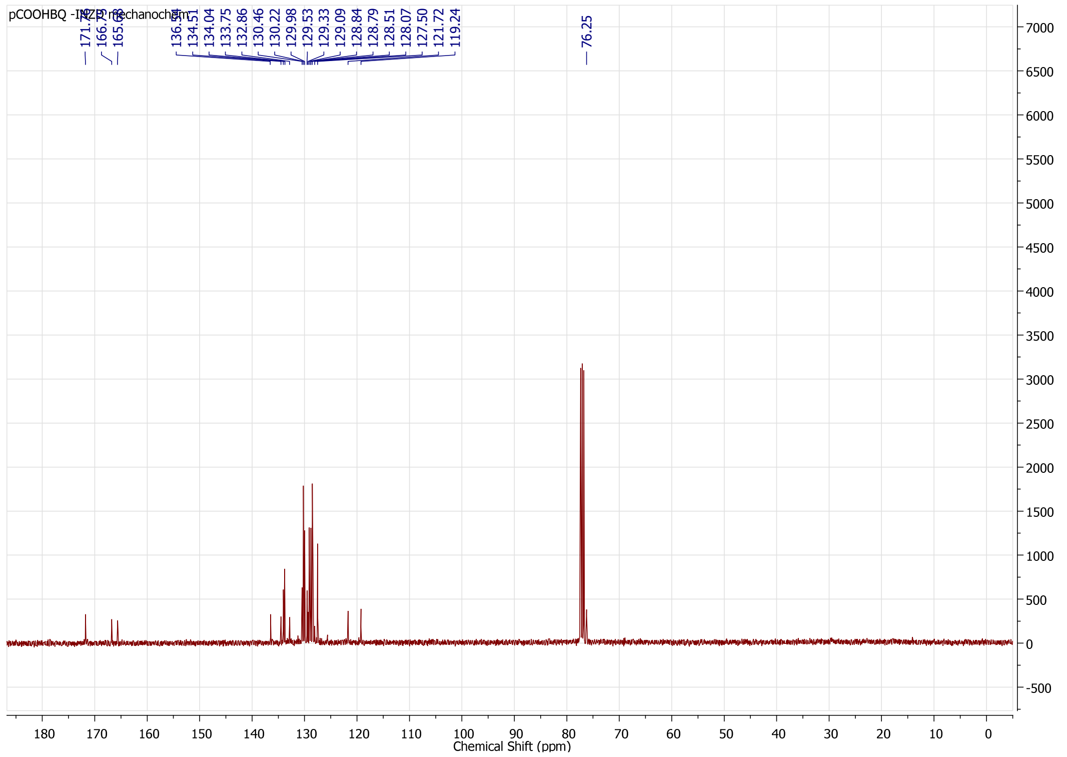

Figure S5.2. ^{13}C NMR (101 MHz, $CDCl_3$) spectrum

E. (3-cyanophenylcarbamoyl)phenyl)methyl benzoate,

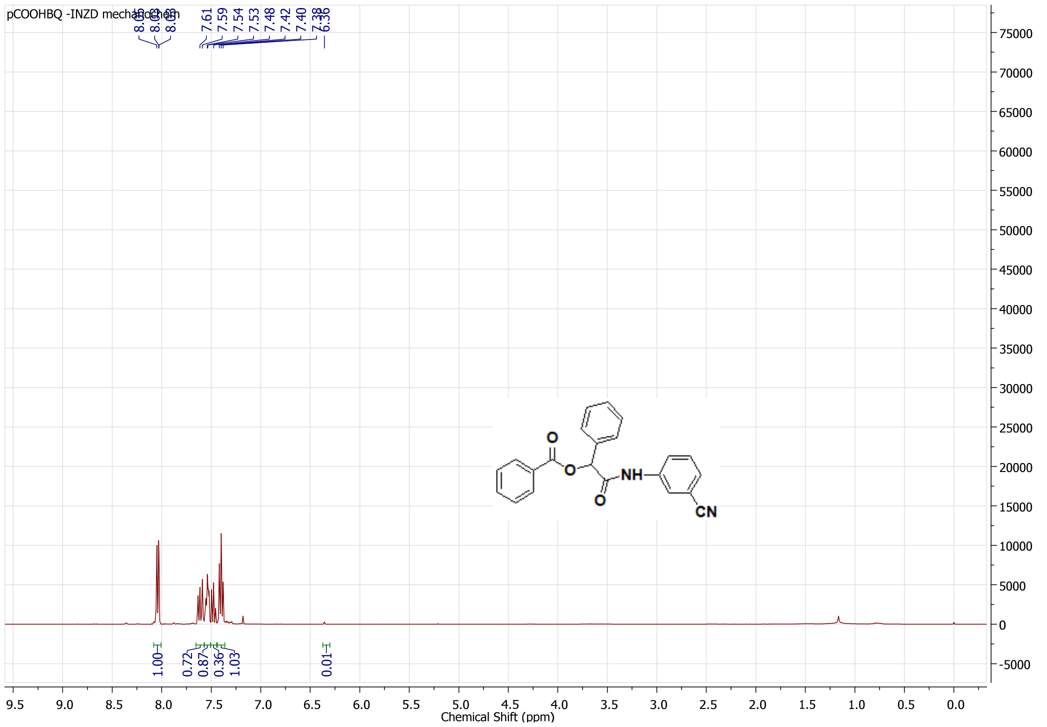

Figure S6.1. ^{1}H NMR (400 MHz, $CDCl_3$) spectrum

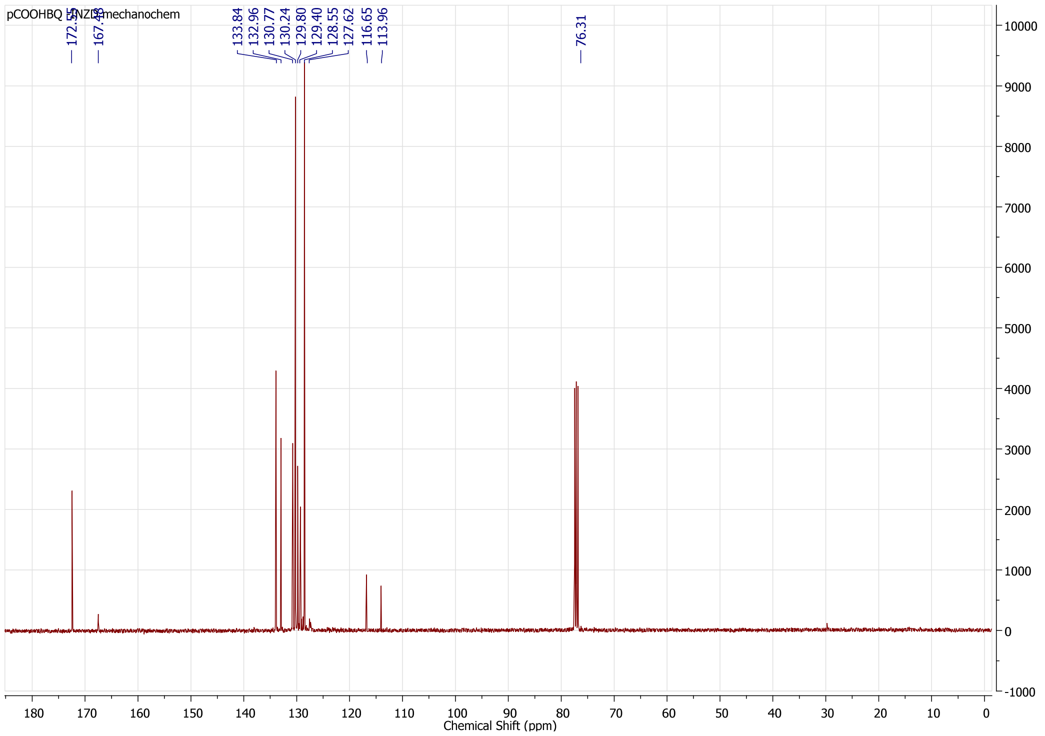

Figure S6.2. ^{13}C NMR (101 MHz, $CDCl_3$) spectrum

F. (*p*-tolycarbamoyl)phenyl)methyl benzoate,

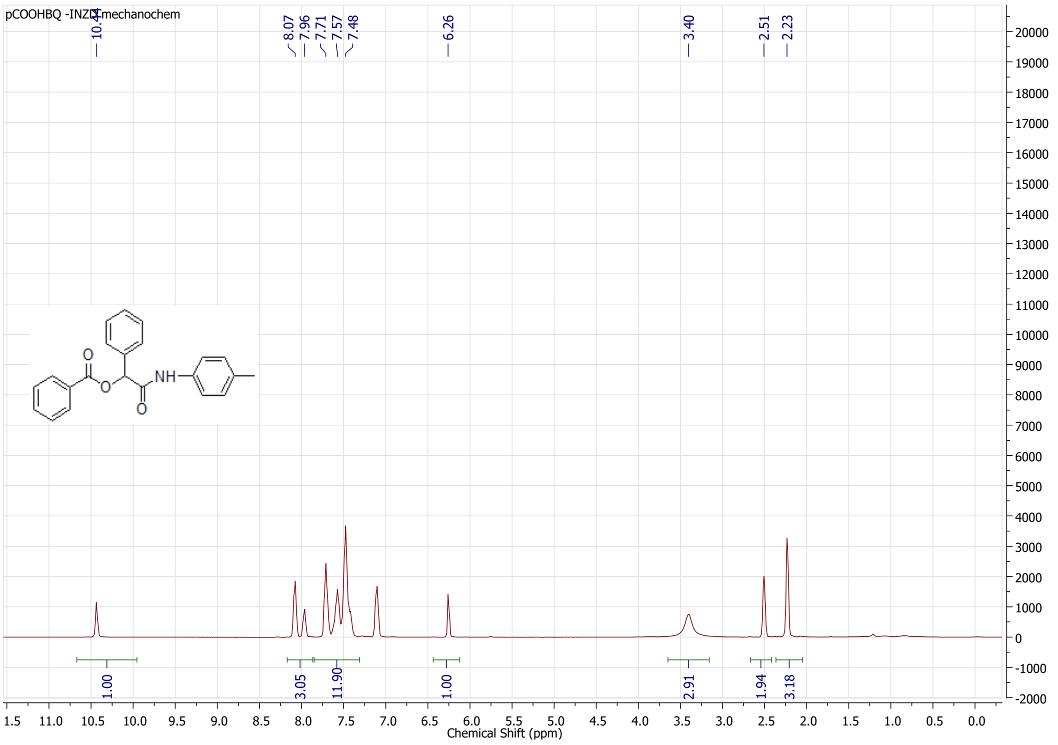

Figure S7.1. ^{1}H NMR (400 MHz, DMSO) spectrum

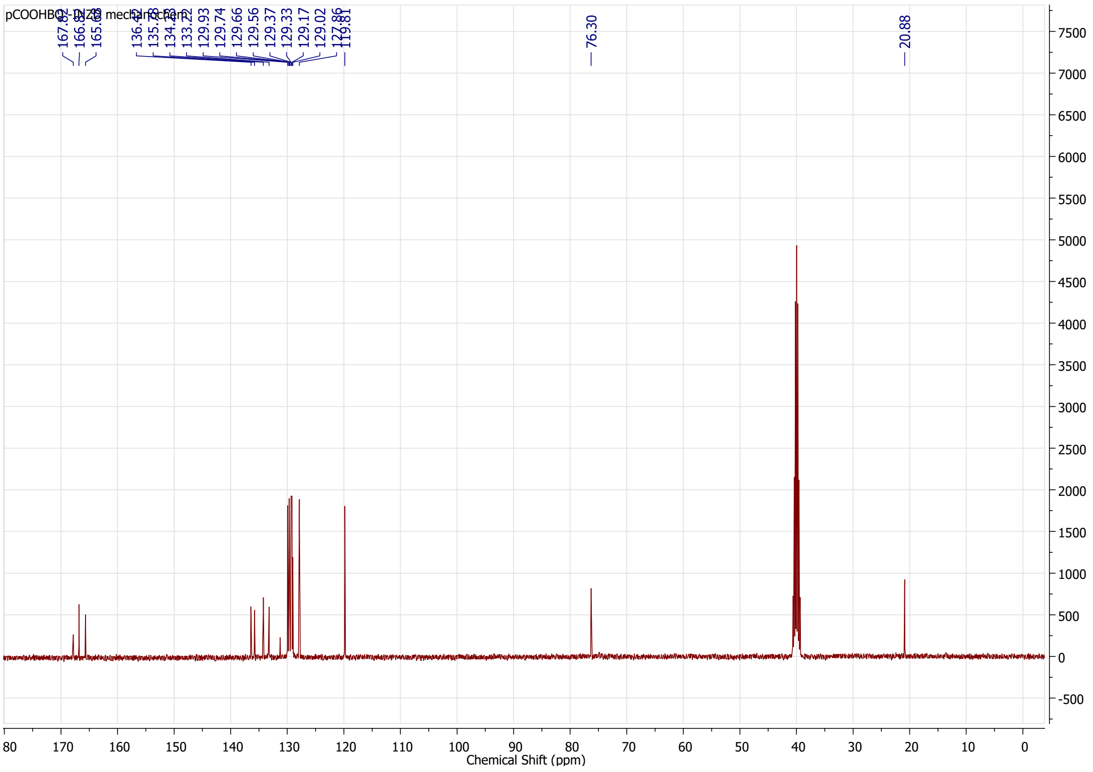

Figure S7.2. ^{13}C NMR (101 MHz, DMSO) spectrum

G. (m-tolylcarbamoyl)(phenyl)methyl benzoate,

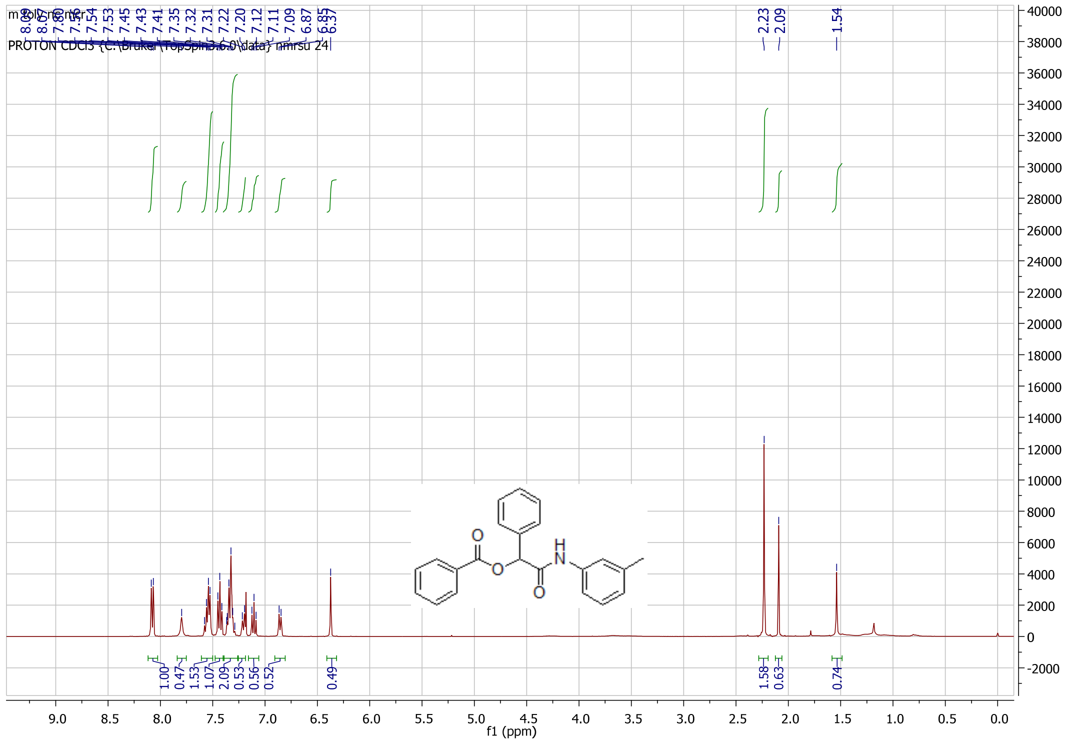

Figure S8.1. ^{1}H NMR (400 MHz, $CDCl_3$) spectrum

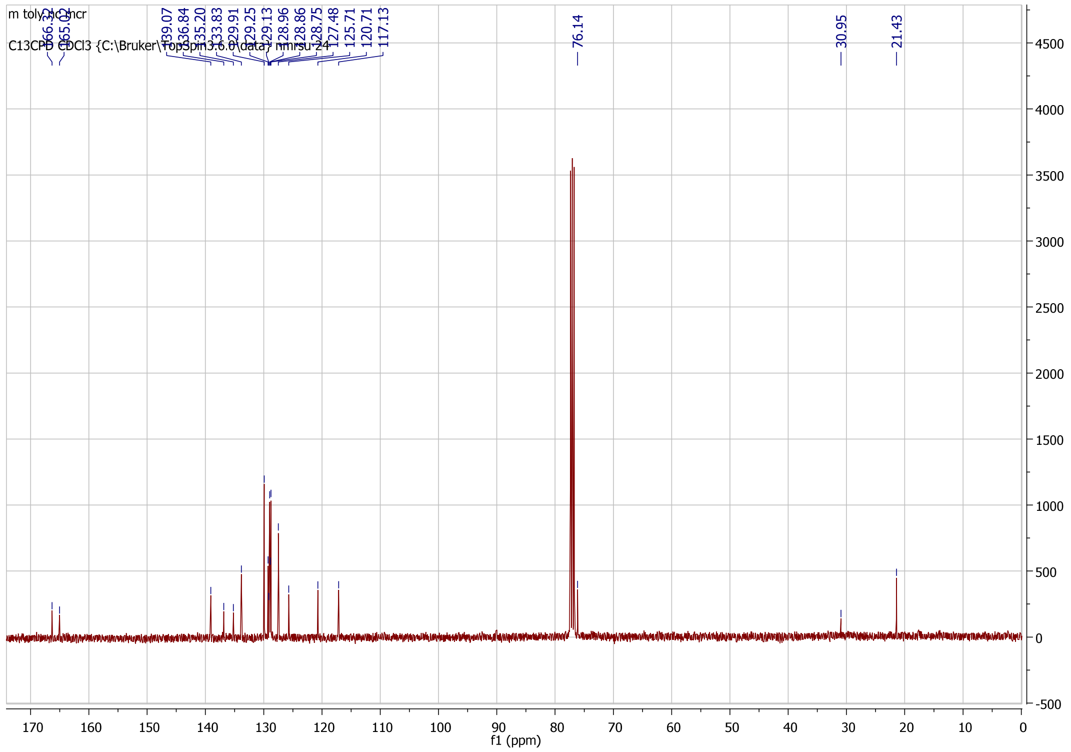

Figure S8.2. ^{13}C NMR (101 MHz, $CDCl_3$) spectrum

H. (3,5-dimethylphenylcarbamoyl)(phenyl)methyl benzoate.

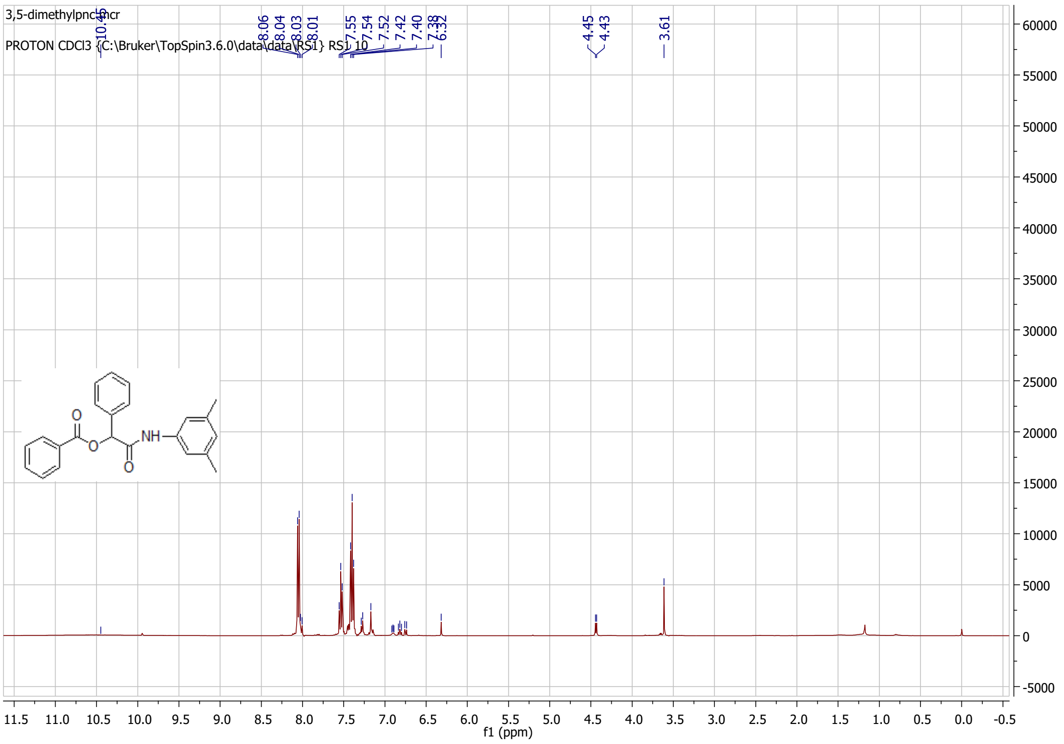

Figure S9.1. ^{1}H NMR (400 MHz, $CDCl_3$) spectrum

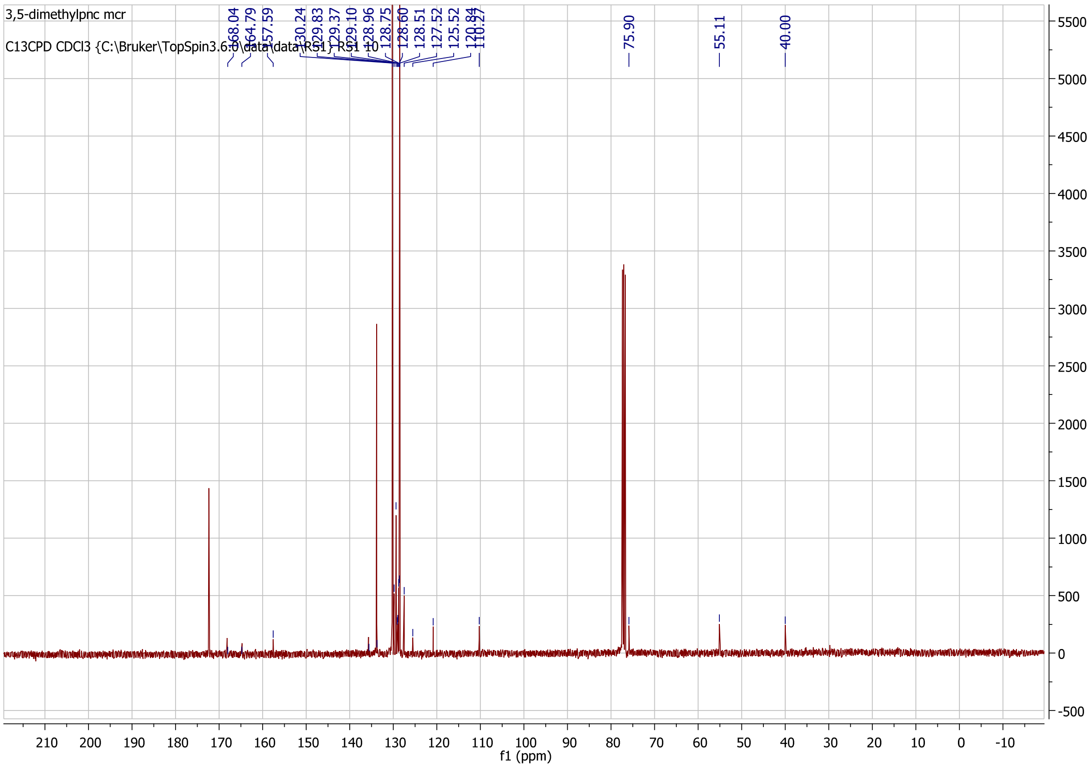

Figure S9.2. ^{13}C NMR (101 MHz, $CDCl_3$) spectrum

I. (mesitylcarbamoyl)(phenyl)methyl benzoate,

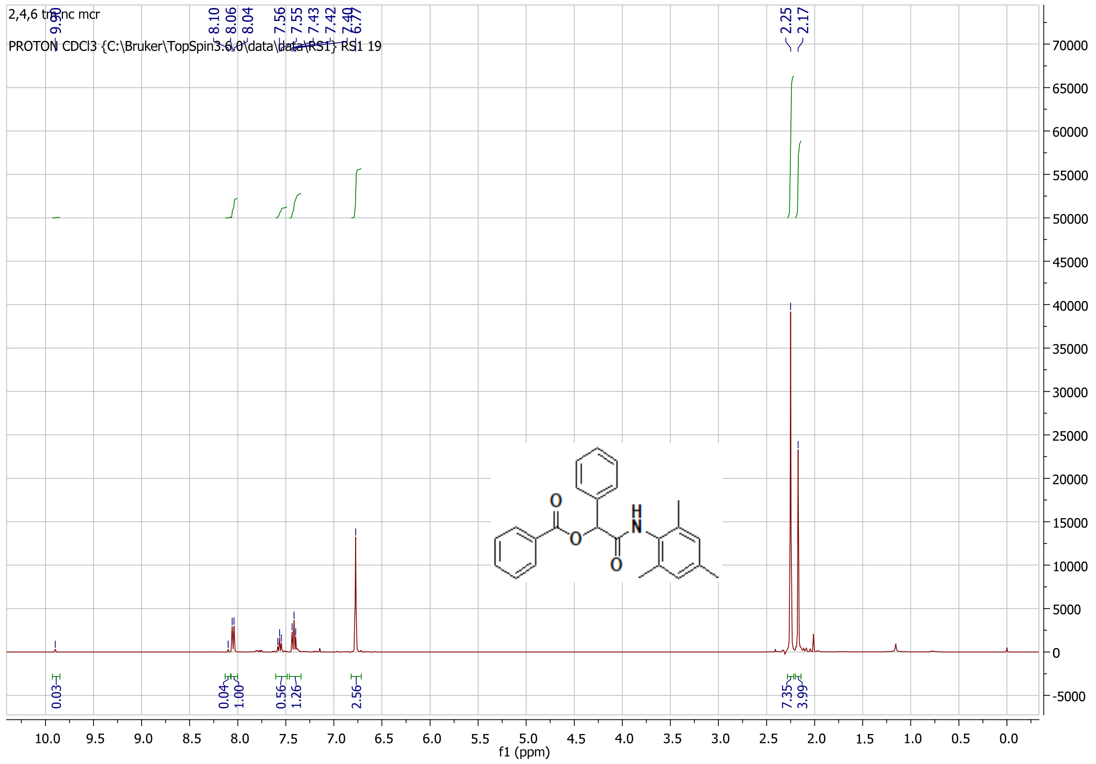

Figure S10.1. ^{1}H NMR (400 MHz, $CDCl_3$) spectrum

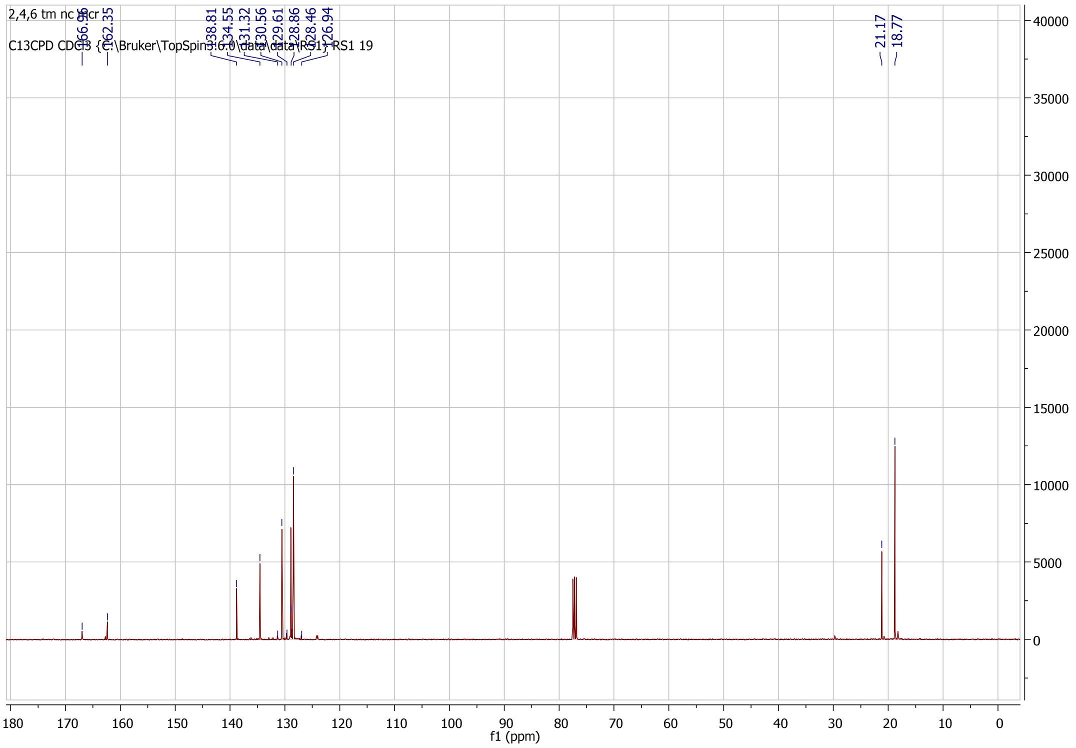

Figure S10.2. ^{13}C NMR (101 MHz, $CDCl_3$) spectrum

J. (4-methyl-2-nitrophenylcarbamoyl)phenyl)methylbenzoate,

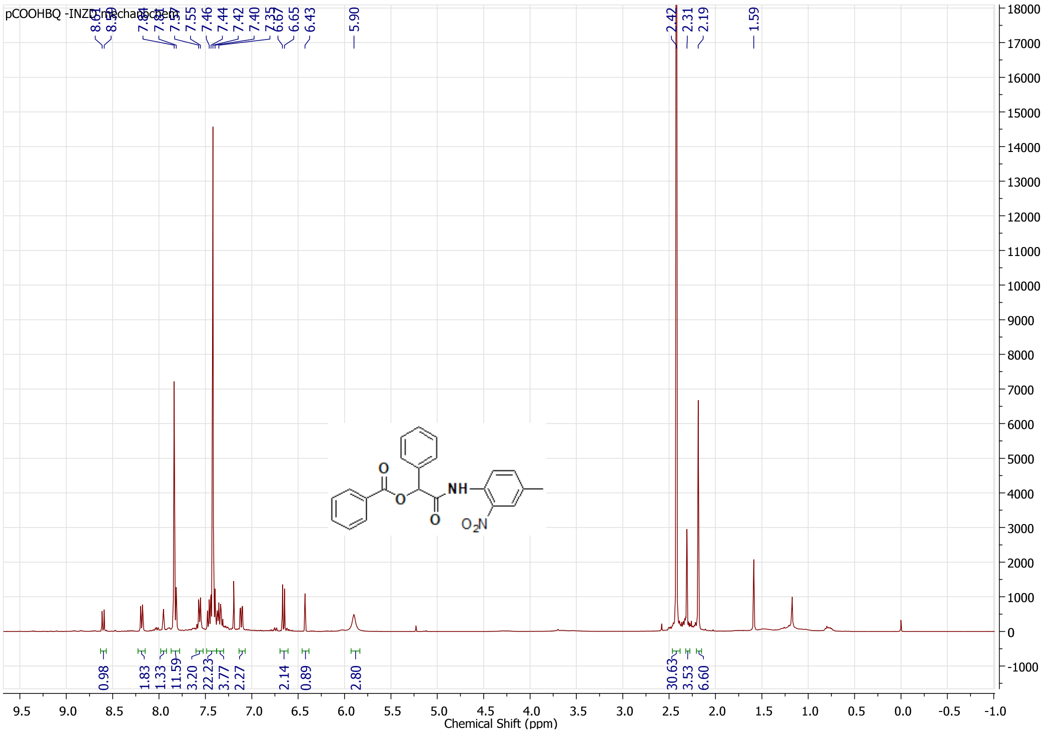

Figure S11.1. ^{1}H NMR (400 MHz, $CDCl_3$) spectrum

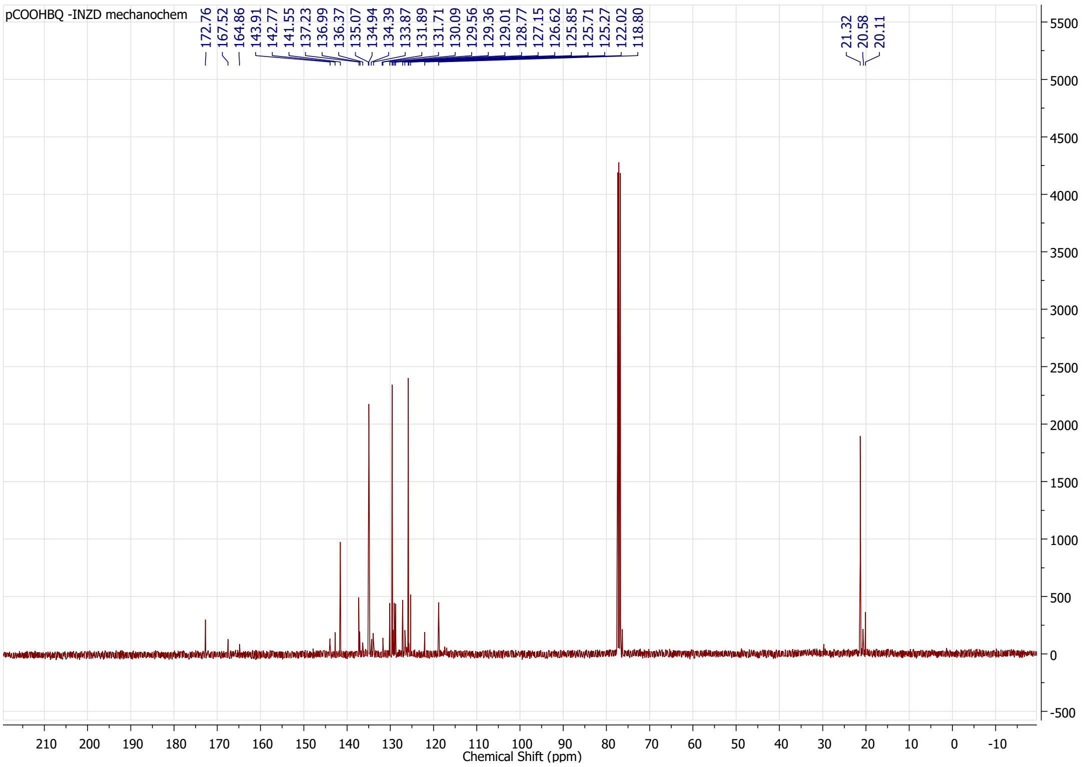

Figure S11.2. ^{13}C NMR (101 MHz, $CDCl_3$) spectrum

K. (4-methoxyphenylcarbamoyl)phenyl)methyl benzoate,

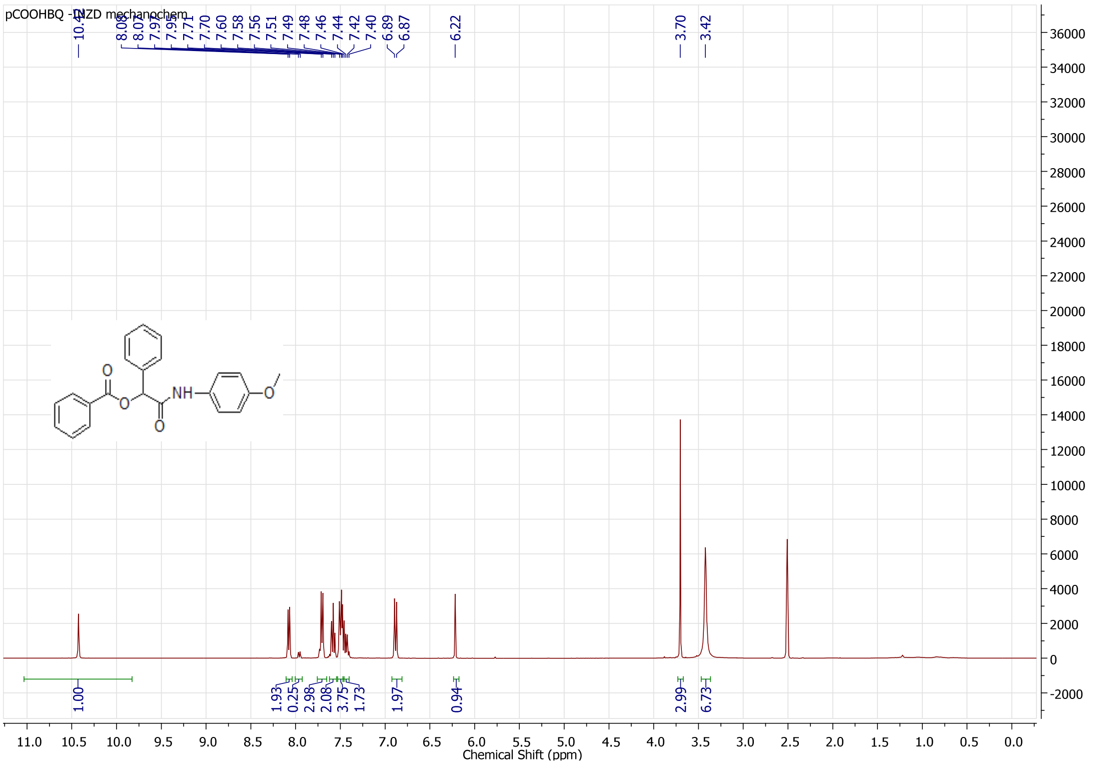

Figure S12.1. ^{1}H NMR (400 MHz, DMSO) spectrum

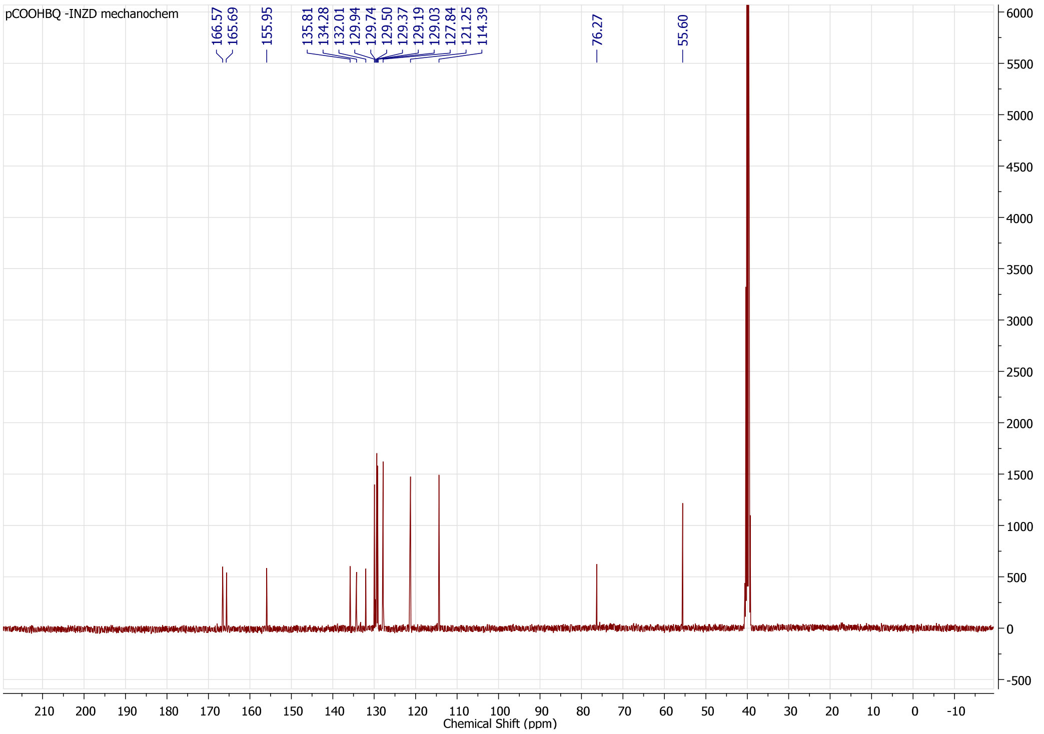

Figure S12.2. ^{13}C NMR (101 MHz, DMSO) spectrum

L. (2-methoxybenzylcarbamoyl)phenyl)methyl benzoate,

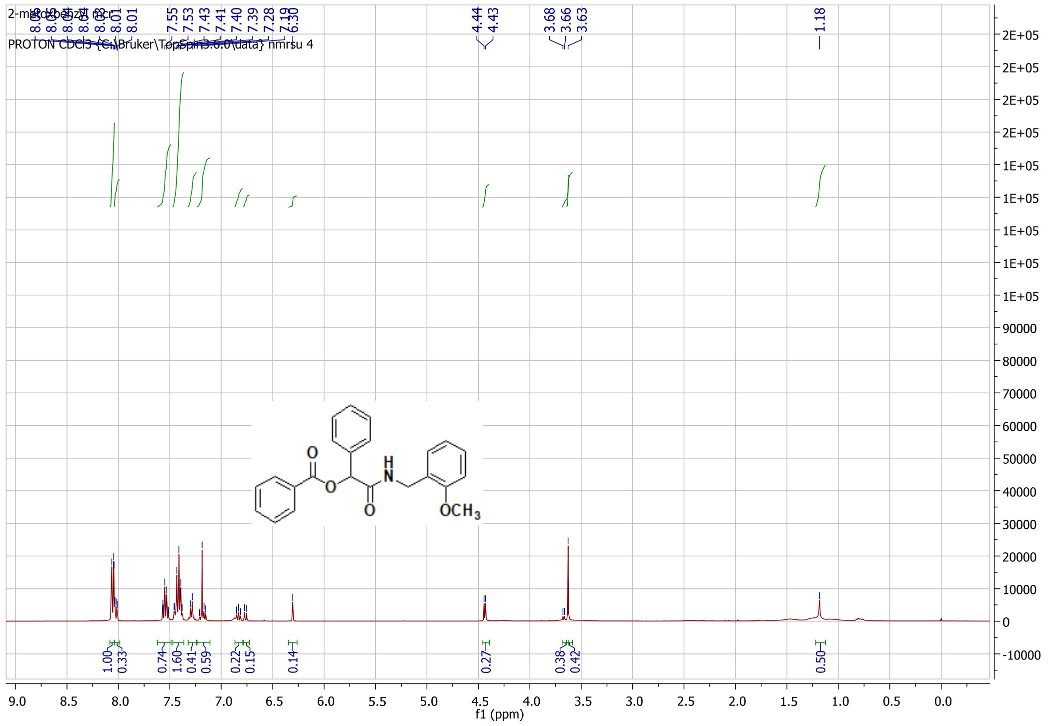

Figure S13.1. ^{1}H NMR (400 MHz, $CDCl_3$) spectrum

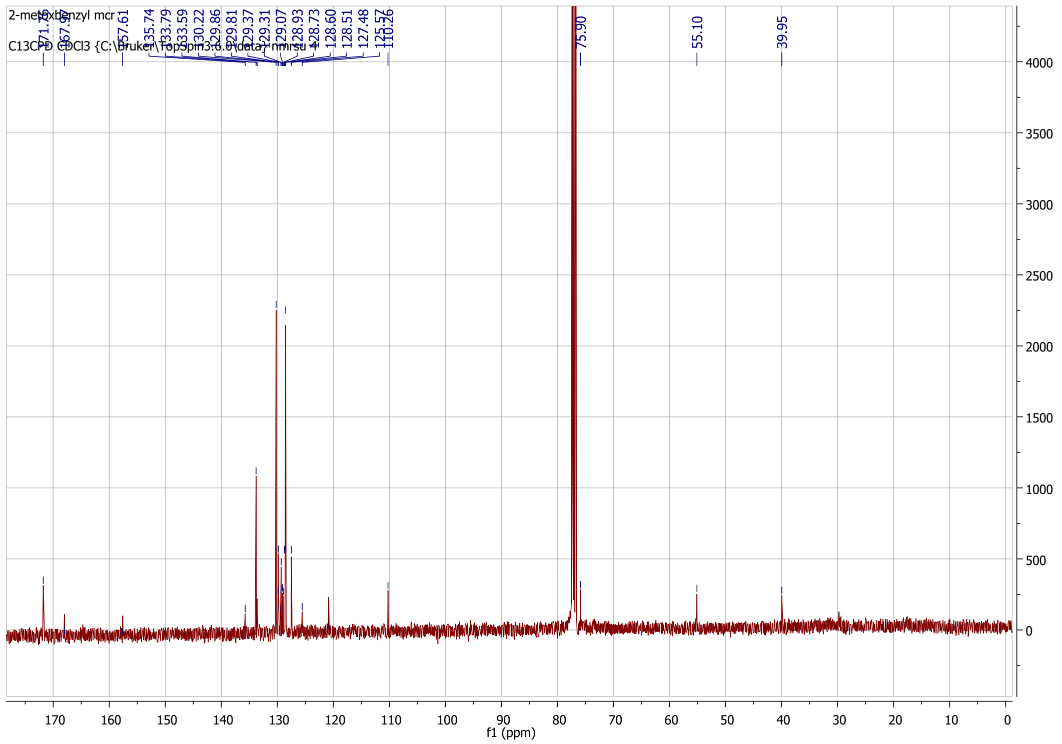

Figure S13.2. ^{13}C NMR (101 MHz, $CDCl_3$) spectrum

M. (4-methoxybenzylcarbamoyl)phenyl)methyl benzoate,

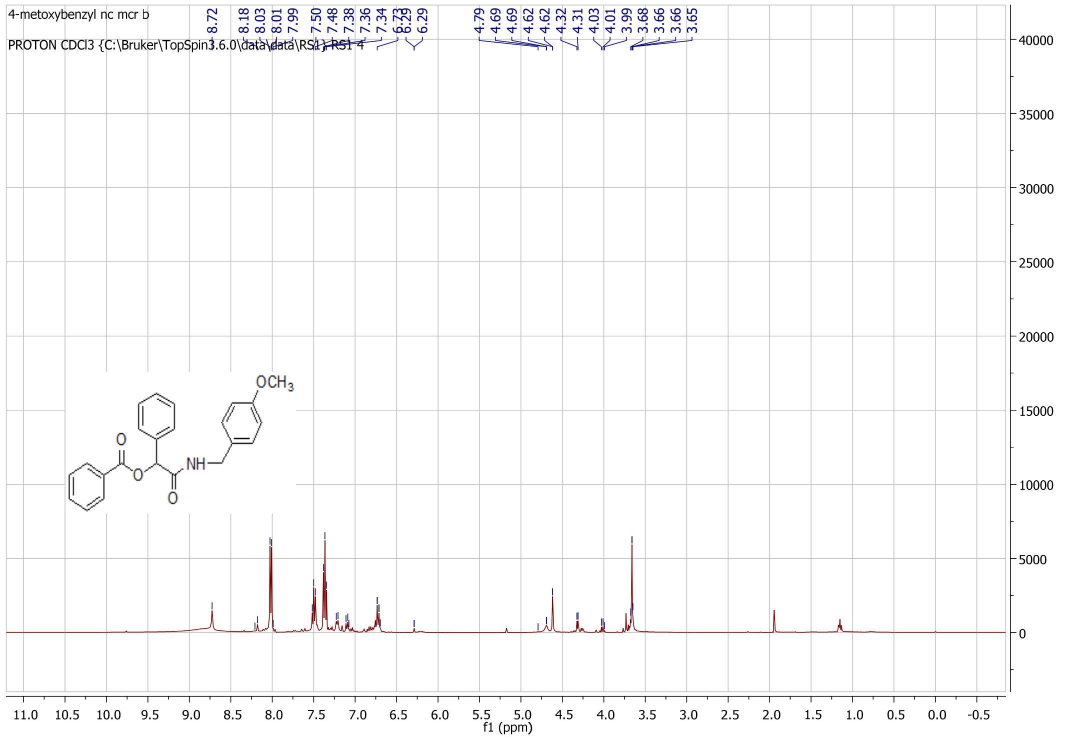

Figure S14.1. ^{1}H NMR (400 MHz, $CDCl_3$) spectrum

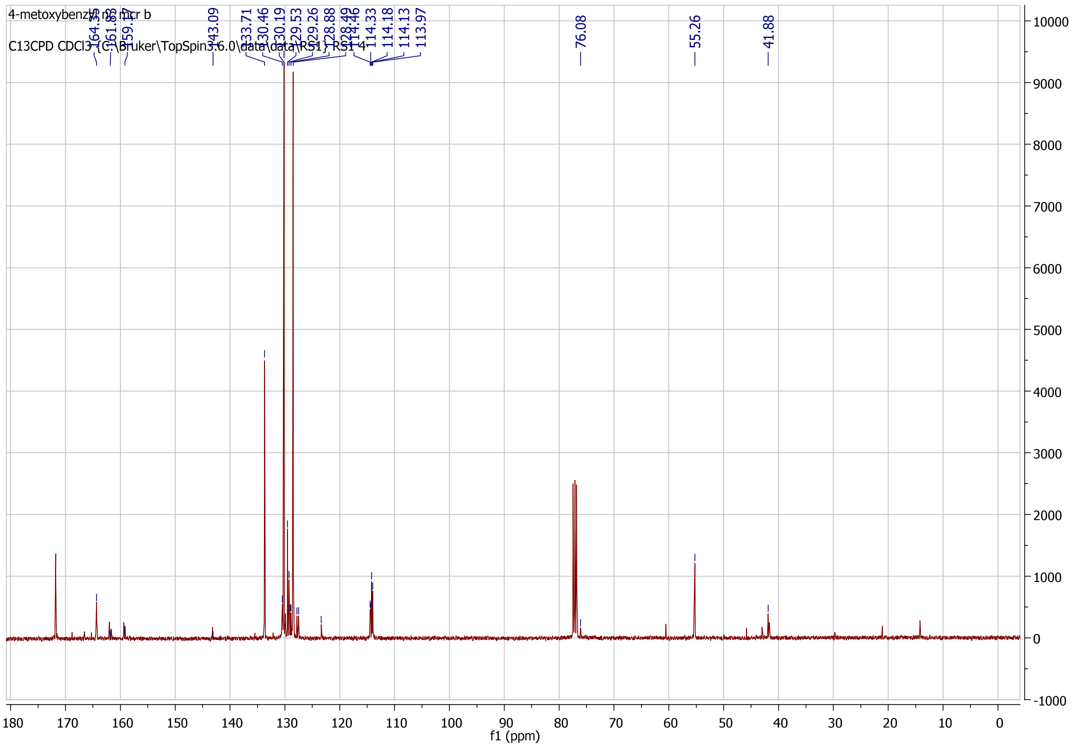

Figure S14.2. ^{13}C NMR (101 MHz, $CDCl_3$) spectrum

N. ethyl 2-[2-(benzoyloxy)-2-phenylacetamido]benzoate,

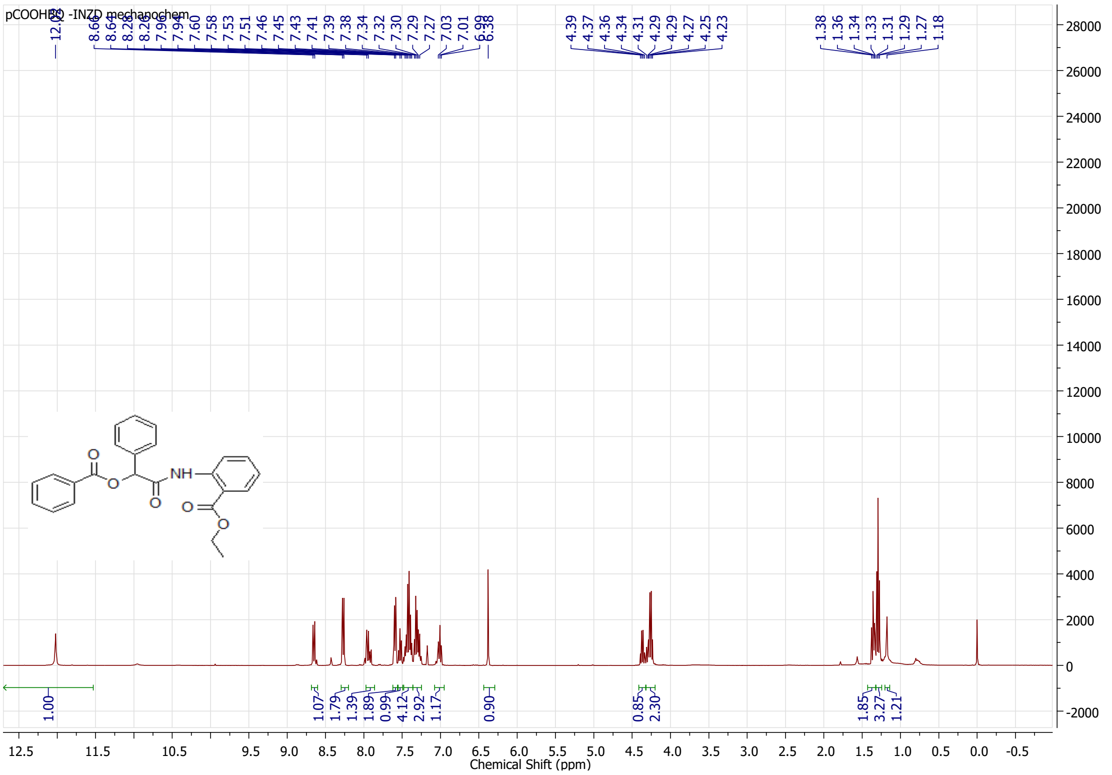

Figure S15.1. ^{1}H NMR (400 MHz, $CDCl_3$) spectrum

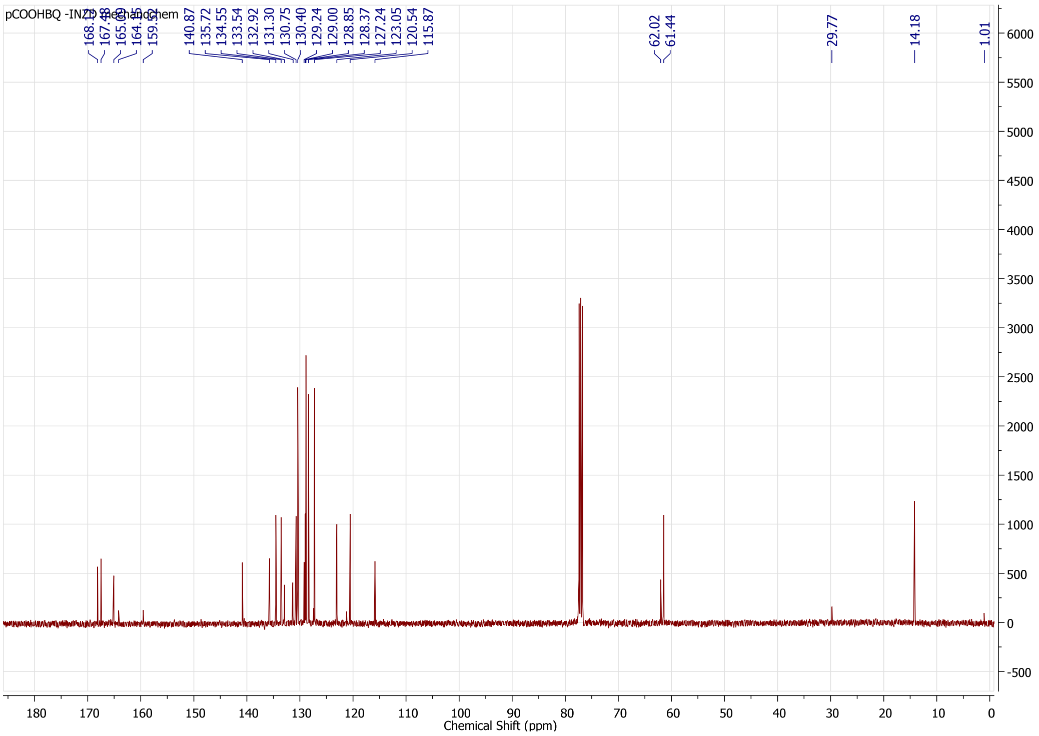

Figure S15.2. ^{13}C NMR (101 MHz, $CDCl_3$) spectrum

O. (naphthalen-3-ylcarbamoyl)(phenyl)methyl benzoate,

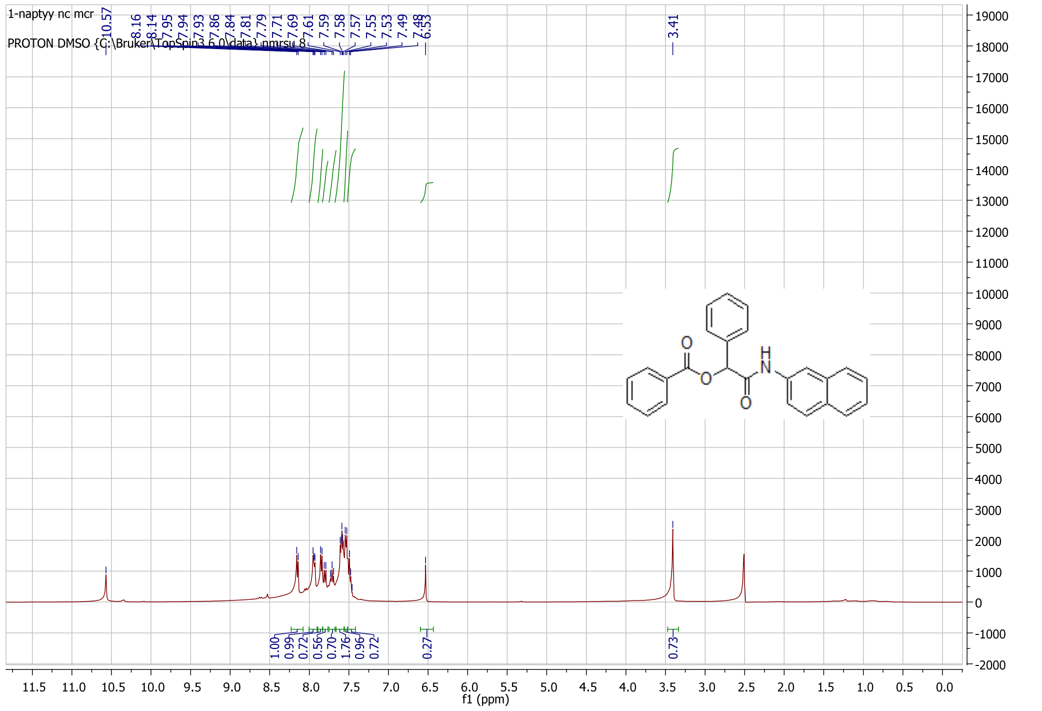

Figure S16.1. ^{1}H NMR (400 MHz, DMSO) spectrum

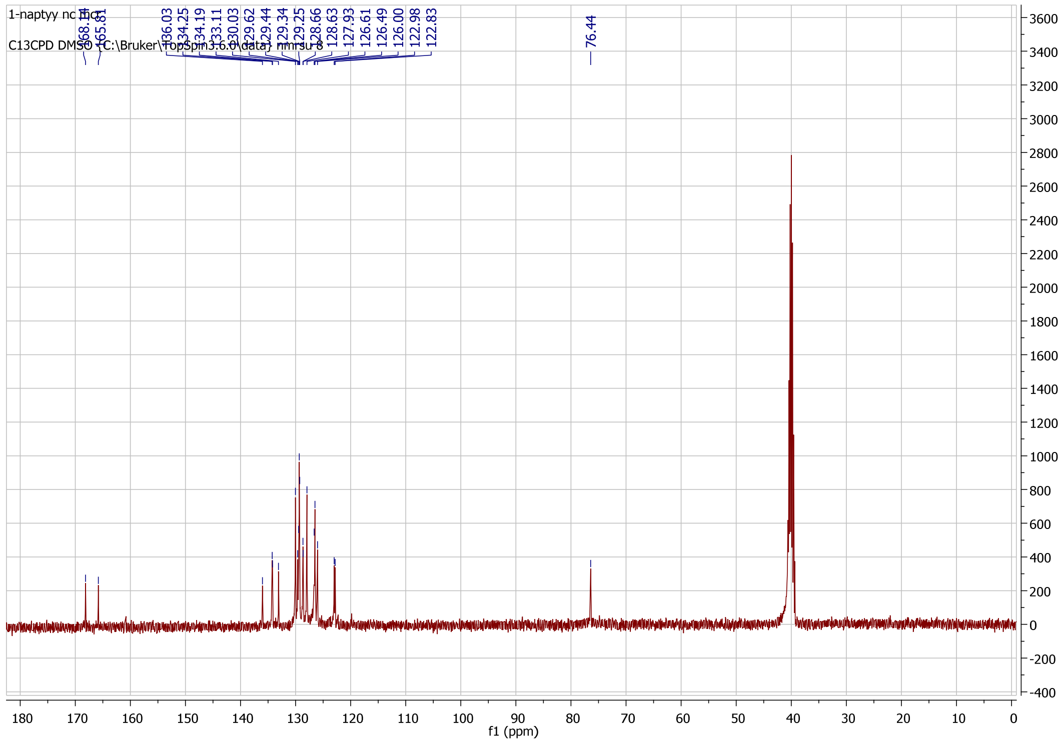

Figure S16.2. ^{13}C NMR (101 MHz, DMSO) spectrum

P. (2-iodophenylcarbamoyl)phenyl)methyl benzoate,

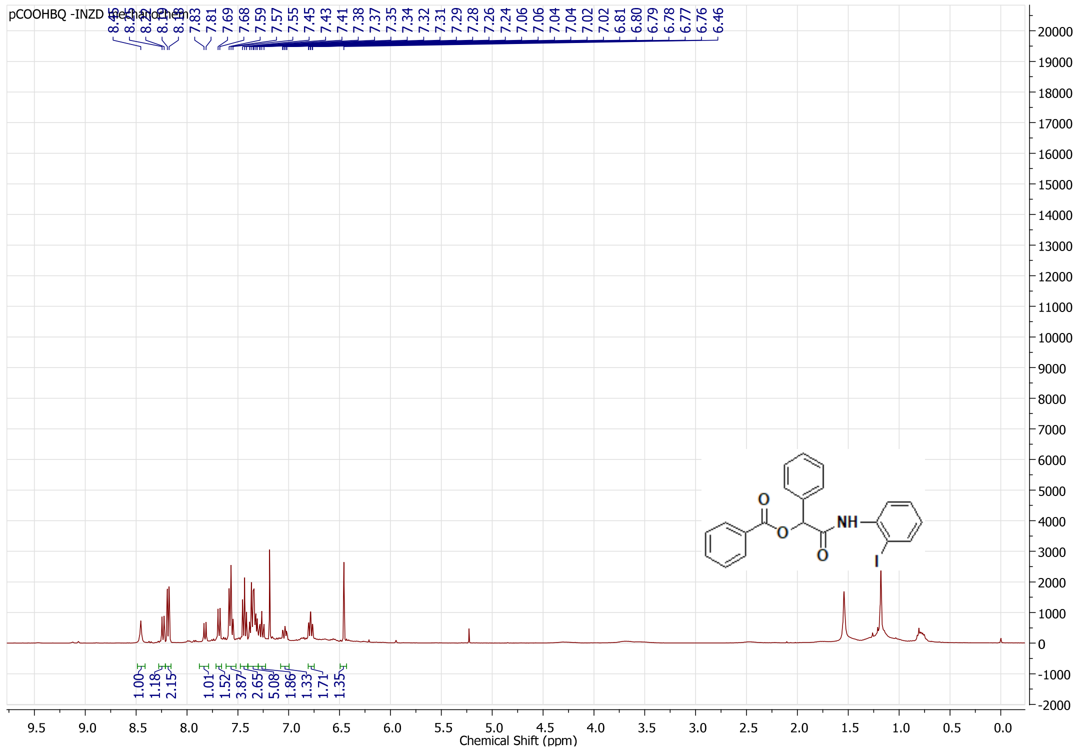

Figure S17.1. ^{1}H NMR (400 MHz, $CDCl_3$) spectrum

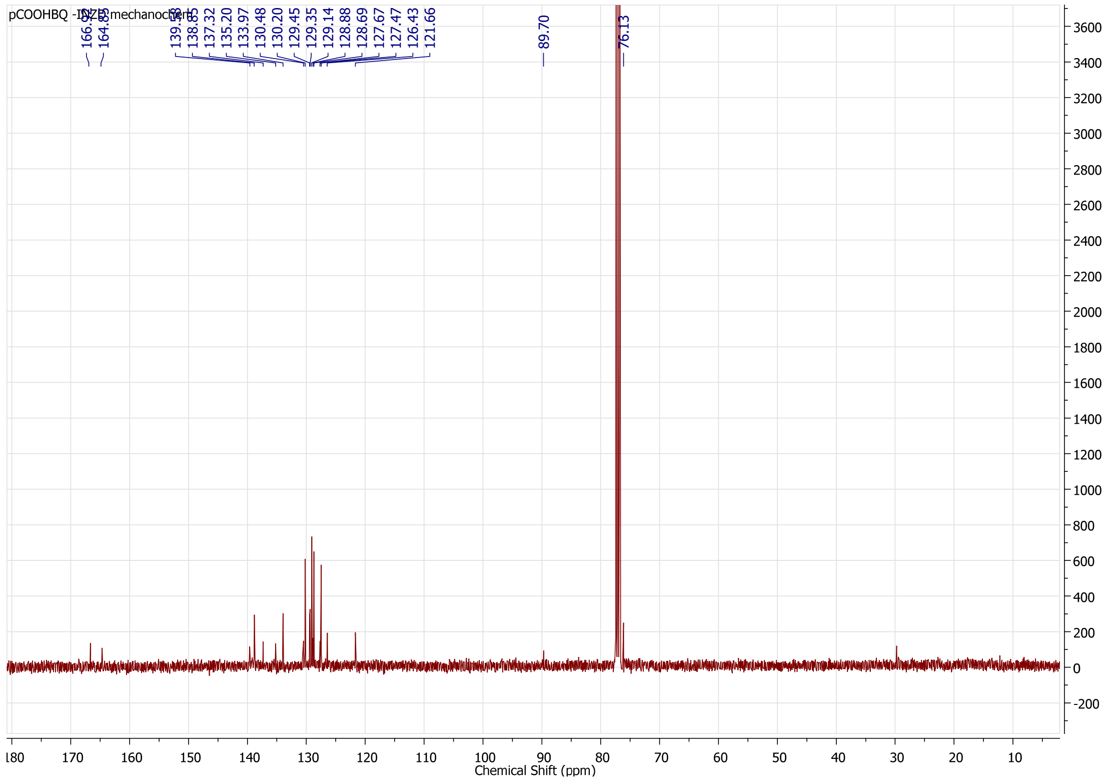

Figure S17.2. ^{13}C NMR (101 MHz, $CDCl_3$) spectrum

Q. (benzo[d]thiazol-2-ylcarbamoyl)(phenyl)methyl benzoate,

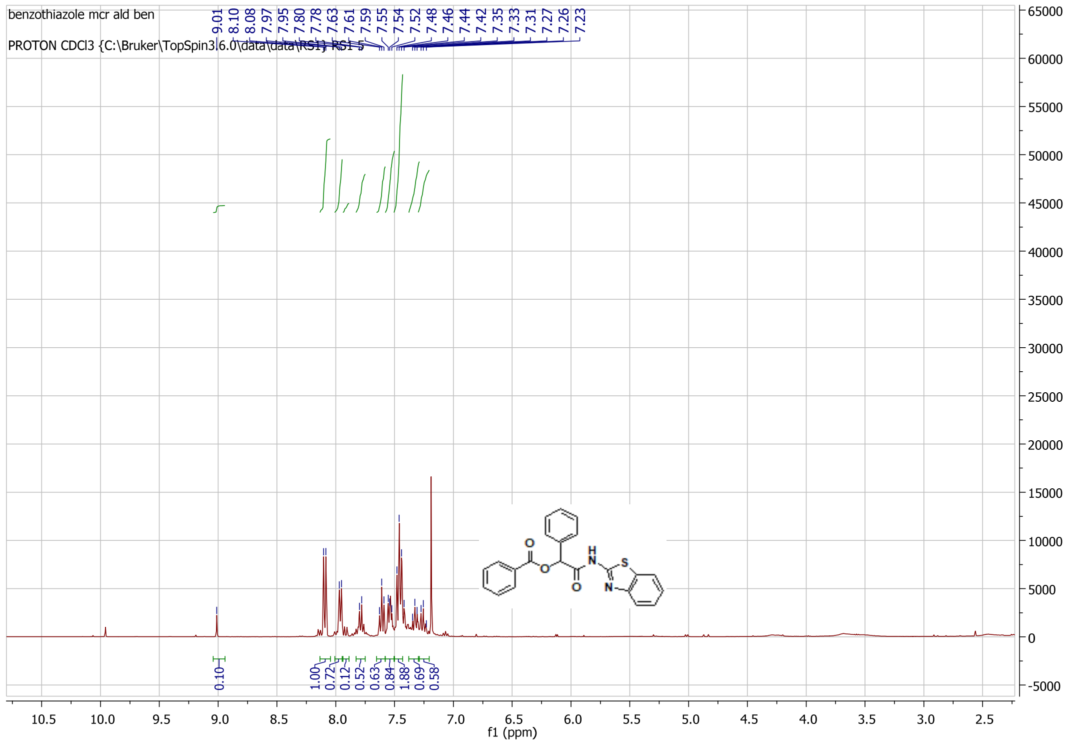

Figure S18.1. ^{1}H NMR (400 MHz, $CDCl_3$) spectrum

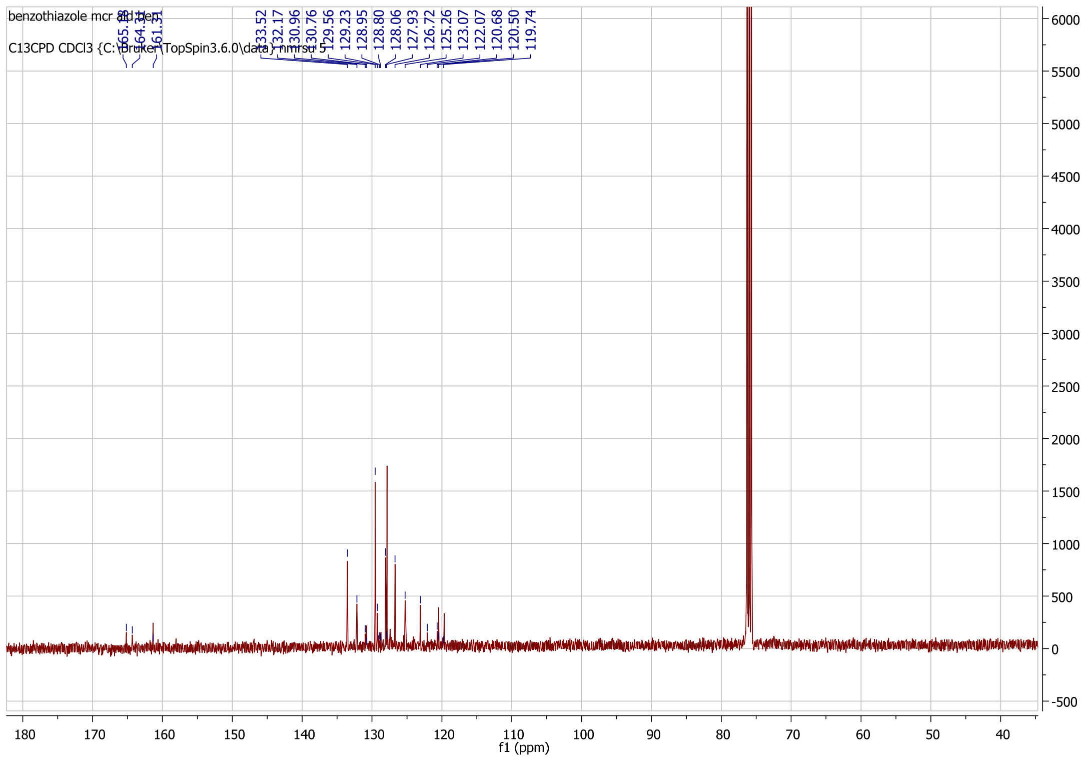

Figure S18.2. ^{13}C NMR (101 MHz, $CDCl_3$) spectrum

R. (3-methoxyphenylcarbamoyl)(2-oxoindolin-3-yl)methyl benzoate.

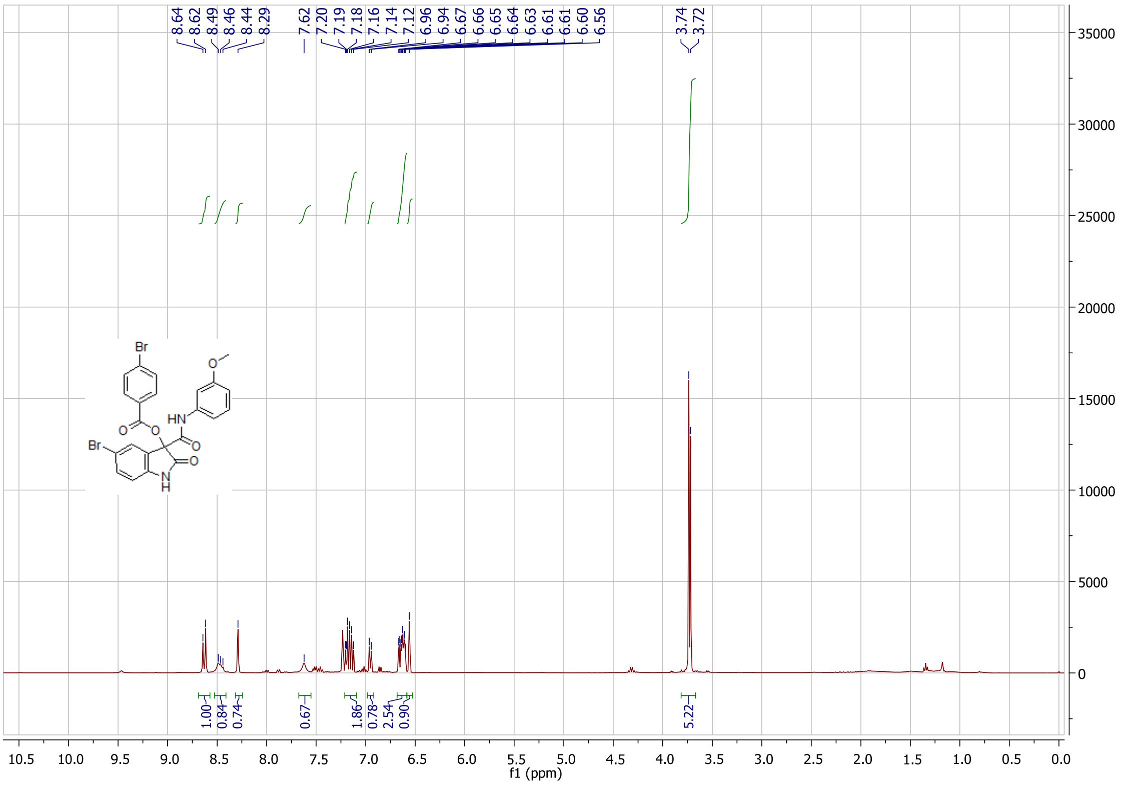

Figure S19.1. ^{1}H NMR (400 MHz, $CDCl_3$) spectrum

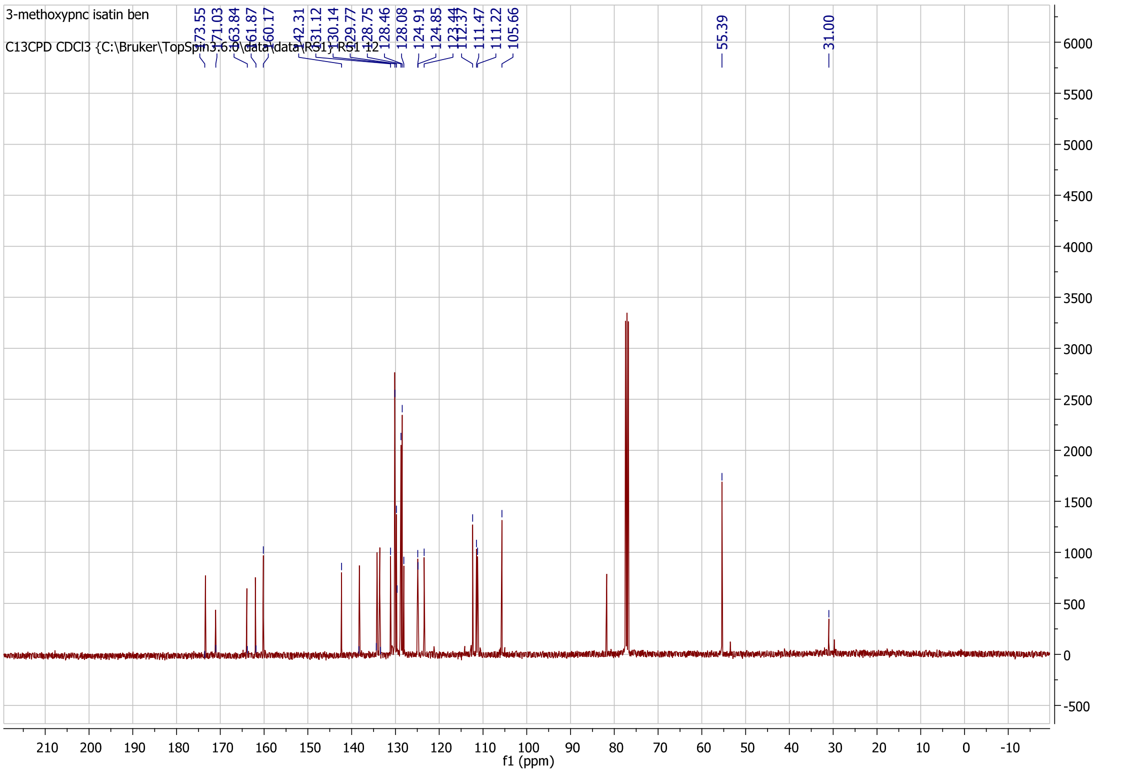

Figure S19.2. ^{13}C NMR (101 MHz, $CDCl_3$) spectrum

T. 3-(p-tolylcarbamoyl)-5-bromo-2-oxoindolin-3-yl 4-bromobenzoate.

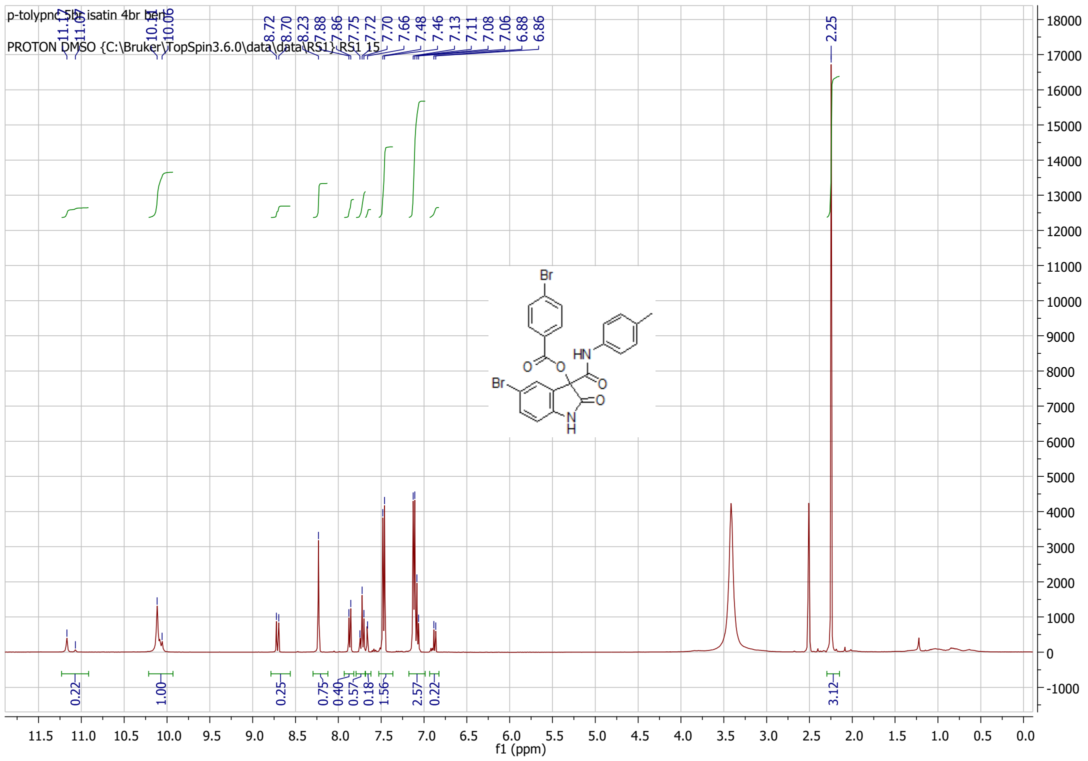

Figure S20.1. ^{1}H NMR (400 MHz, DMSO) spectrum

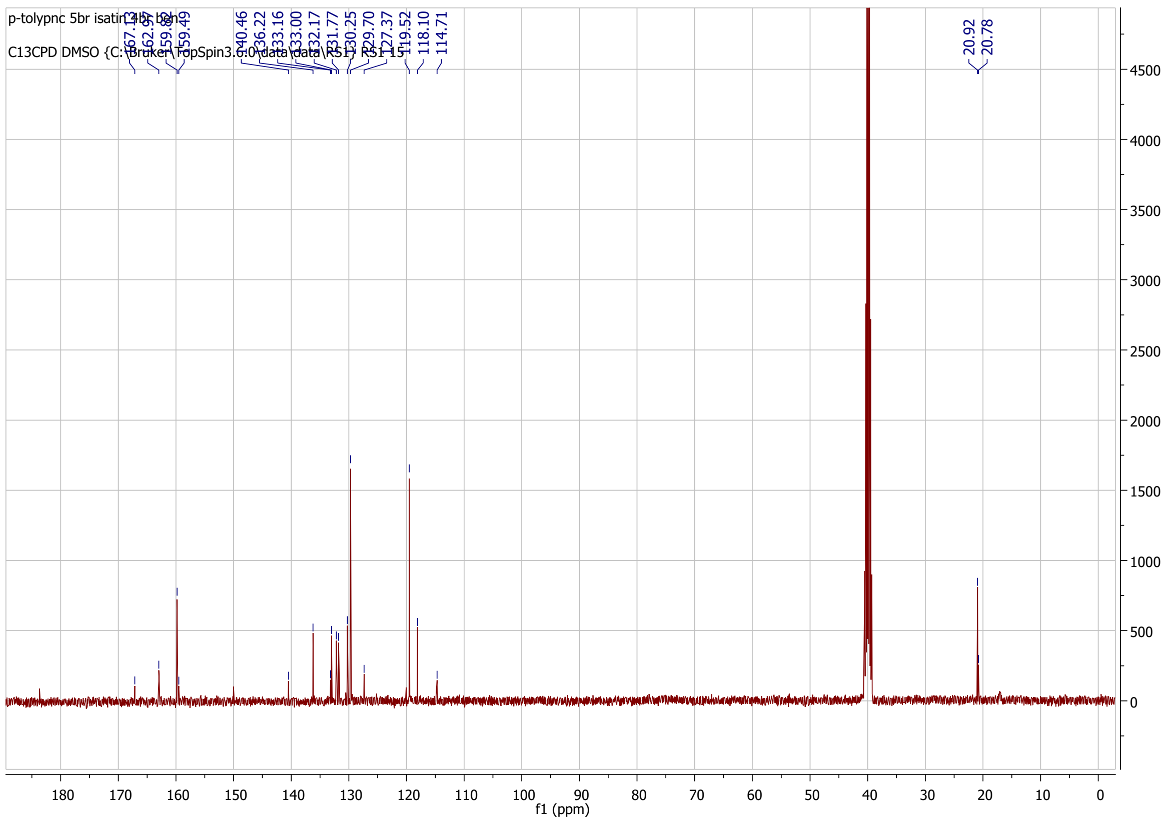

Figure S20.2. ^{13}C NMR (101 MHz, DMSO) spectrum

U. 3-(2-nitrophenylcarbamoyl)-5-bromo-2-oxoindolin-3-yl-4-bromobenzoate

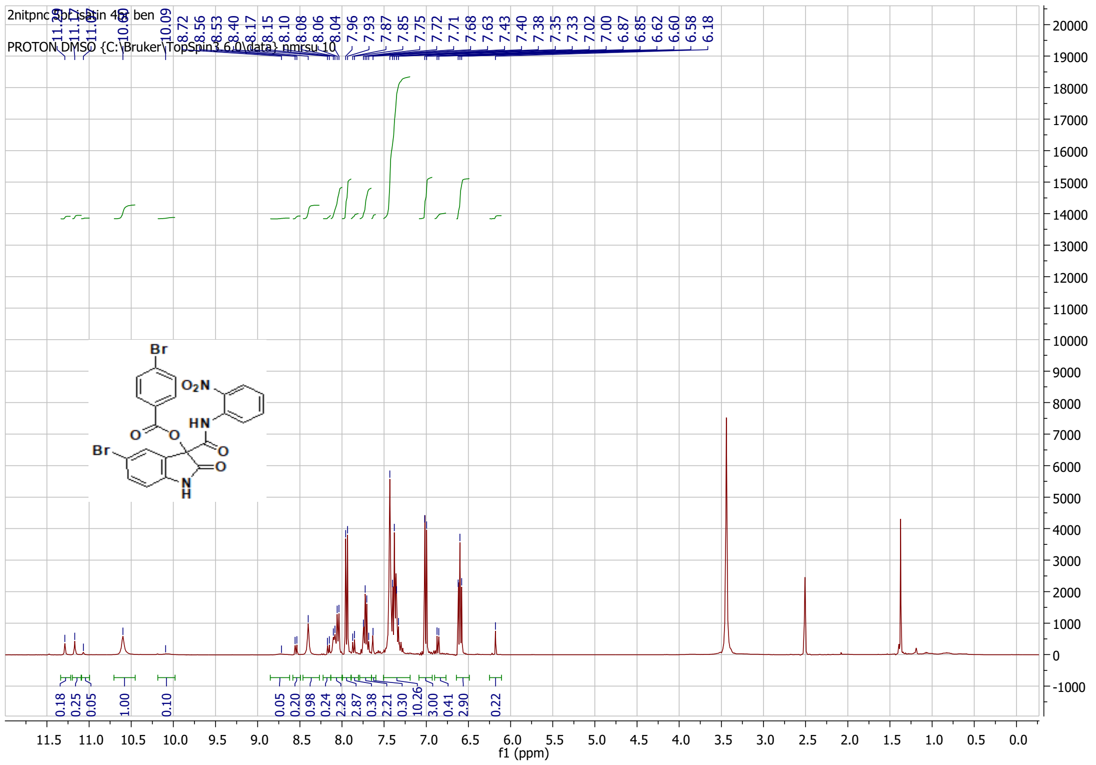

Figure S21.1. ^{1}H NMR (400 MHz, DMSO) spectrum

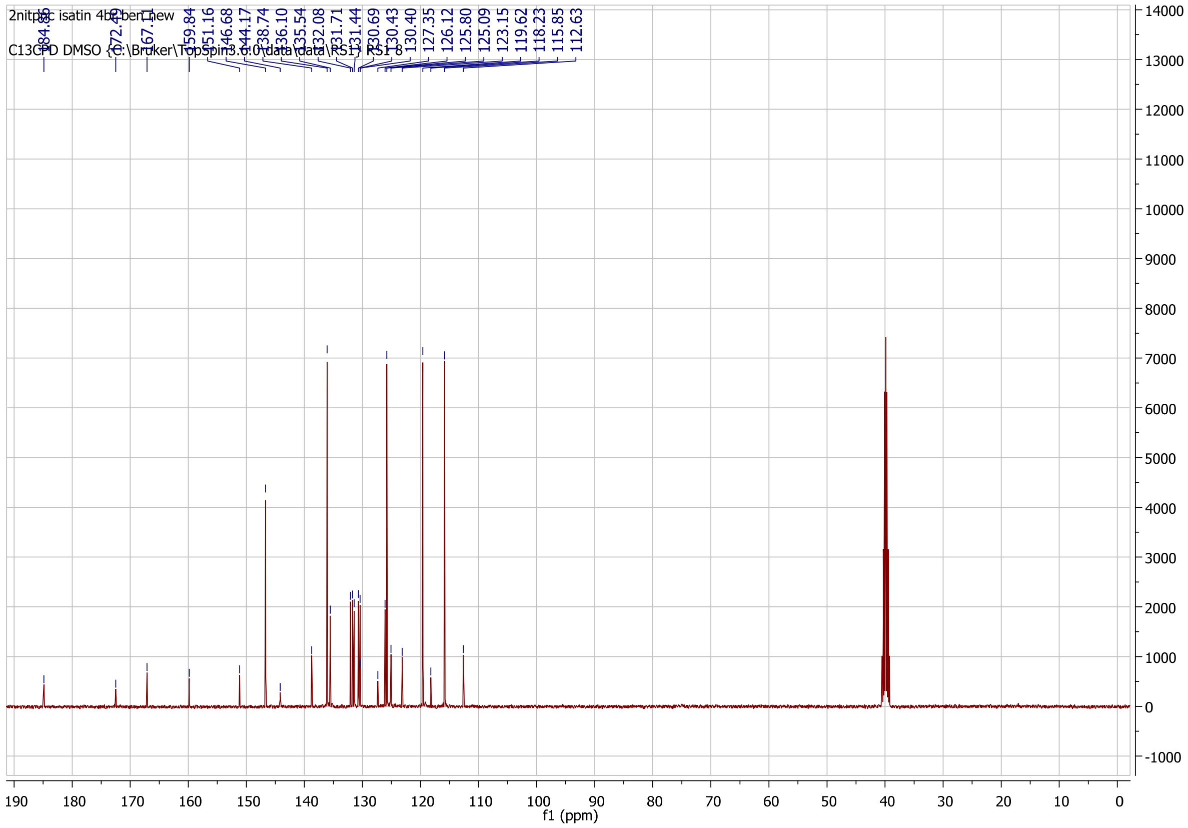

Figure S21.2. ^{13}C NMR (101 MHz, DMSO) spectrum

V. 3-(4-chlorophenoxy)-N-(naphthalen-1-yl)-2-oxoindoline-3-carboxamide

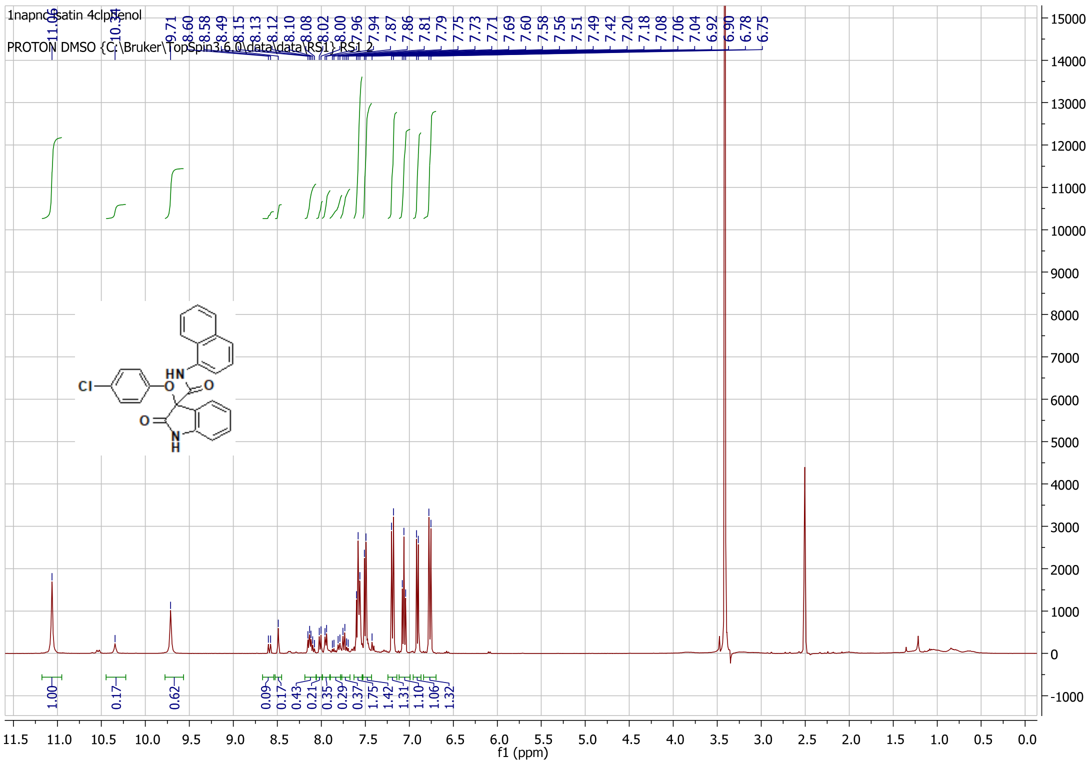

Figure S22.1. ^{1}H NMR (400 MHz, DMSO) spectrum

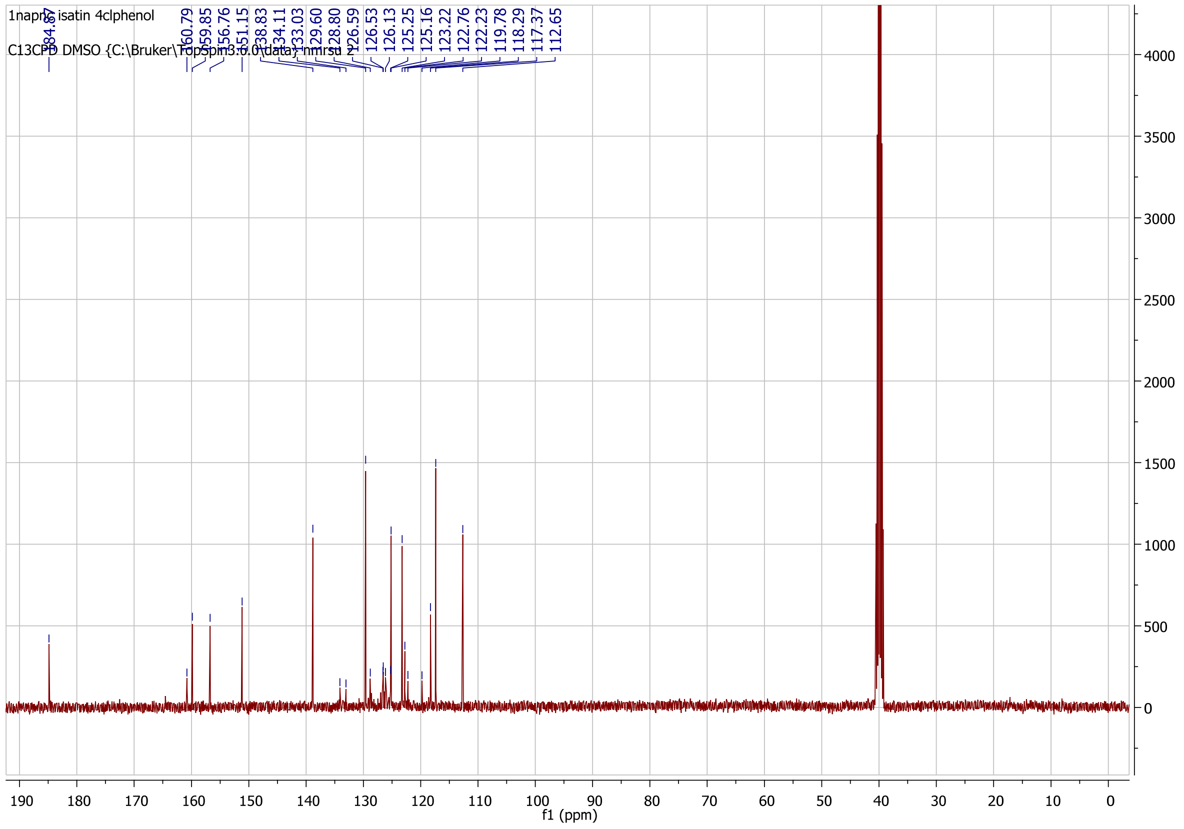

Figure S22.2. ^{13}C NMR (101 MHz, DMSO) spectrum

W. 3-(4-chlorophenoxy)-2-oxo-N-p-tolylindoline-3-carboxamide

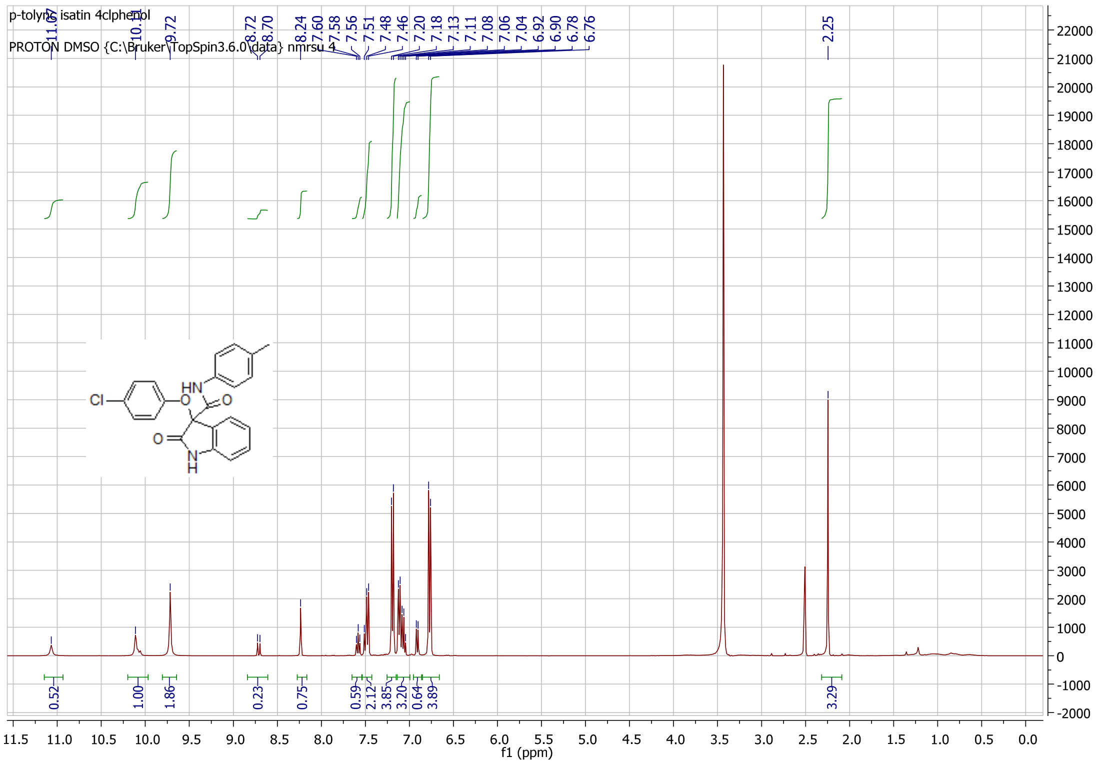

Figure S23.1. ^{1}H NMR (400 MHz, DMSO) spectrum

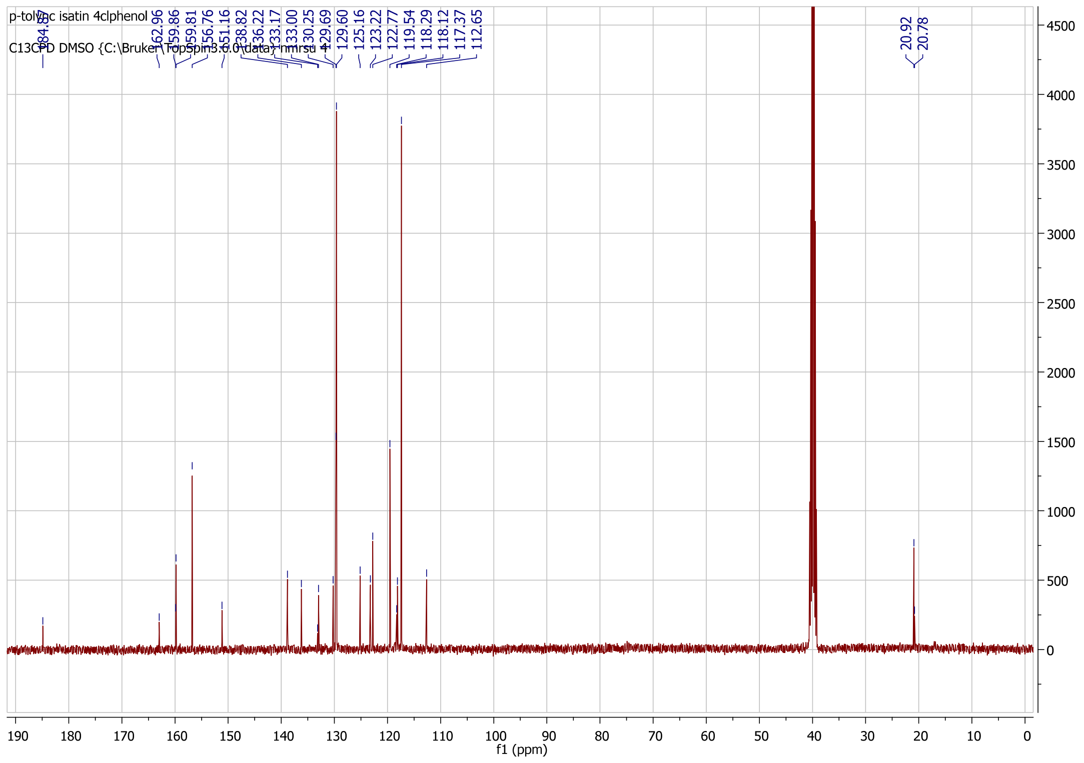

Figure S23.2. ^{13}C NMR (101 MHz, DMSO) spectrum

X. 3-(4-chlorophenoxy)-N-(3,5-dimethylphenyl)-2-oxoindoline-3-carboxamide

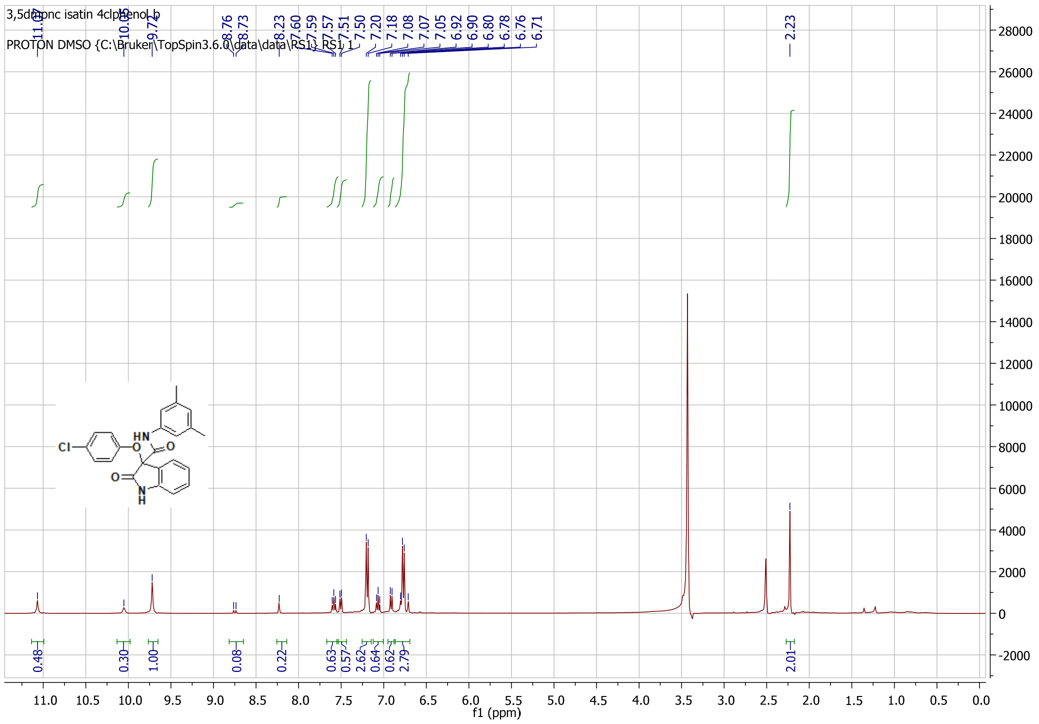

Figure S24.1. 1 ^{1}H NMR (400 MHz, DMSO) spectrum

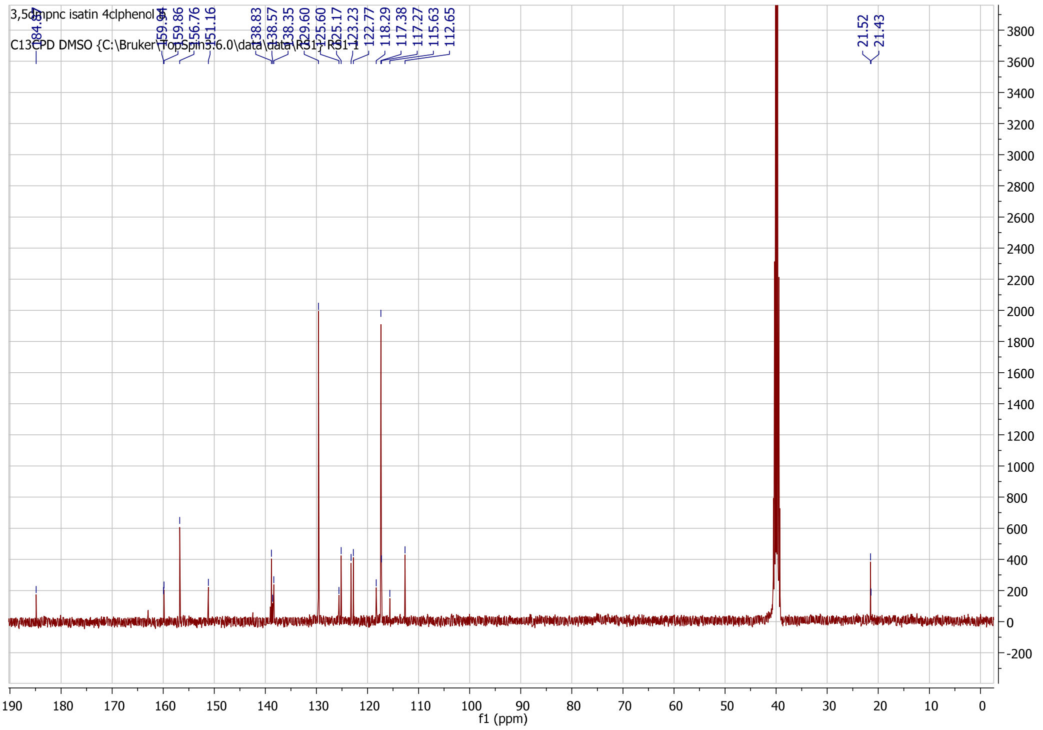

Figure S24.2. ^{13}C NMR (101 MHz, DMSO) spectrum
